# Ambient intelligence governance review: from service-oriented to self-service

**DOI:** 10.7717/peerj-cs.788

**Published:** 2022-01-11

**Authors:** Victor Ponce, Bessam Abdulrazak

**Affiliations:** Ambient Intelligence Lab (AMI-Lab), Université de Sherbrooke, Sherbrooke, Quebec, Canada

**Keywords:** Ambient intelligence, SOA, Service-oriented, Self-service, Service governance, Context-aware

## Abstract

The current generation of connected devices and the Internet of Things augment people’s capabilities through ambient intelligence. Ambient Intelligence (AmI) support systems contain applications consuming available services in the environment to serve users. A well-known design of these applications follows a service architecture style and implement artificial intelligence mechanisms to maintain an awareness of the context: The service architecture style enables the distribution of capabilities and facilitates interoperability. Intelligence and context-awareness provide an adaptation of the environment to improve the interaction. Smart objects in distributed deployments and the increasing machine awareness of devices and people context also lead us to architectures, including self-governed policies providing self-service. We have systematically reviewed and analyzed ambient system governance considering service-oriented architecture (SOA) as a reference model. We applied a systematic mapping process obtaining 198 papers for screening (out of 712 obtained after conducting searches in research databases). We then reviewed and categorized 68 papers related to 48 research projects selected by fulfilling ambient intelligence and SOA principles and concepts. This paper presents the result of our analysis, including the existing governance designs, the distribution of adopted characteristics, and the trend to incorporate service in the context-aware process. We also discuss the identified challenges and analyze research directions.

## Introduction

Ambient intelligence (AmI) support systems are a suitable element in the augmentation of people’s capabilities (Sadri, 2011; Acampora et al., 2013). These systems perform a smart use of the context in our environment by taking advantage of the technology-enriched surroundings to support users (Augusto & Mccullagh, 2007) in a context-aware manner. When systems require the interaction and integration of internal and external entities, ambient systems tightly adopt Service-Oriented Computing (SOC) (Stavropoulos, Vrakas & Vlahavas, 2011). SOC is a computing paradigm that considers services the principal concern when modeling and implementing applications (Papazoglou, 2003; Papazoglou et al., 2008). SOC includes a Service-Oriented Architecture (SOA) style, which defines a set of principles and design patterns. SOA is the reference model in SOC, introducing an abstraction of the solution logic: services invoked by consumers in a platform. SOC and SOA elements facilitate the interoperability and governance of services (Erl, 2008) by having well-deployed ambient support systems (Urbieta et al., 2008; Stavropoulos, Vrakas & Vlahavas, 2011). A category of AmI solutions also incorporates agents following SOA. The agents implement a pervasive empathy context, decreasing technology overload on users using services and applications (Maes, 1994).

Applications in AmI incorporate and intelligently utilize existing context information through sensor devices and interact with the environment through actuator devices, *e.g*., an assistive smart home application for health care supporting elderly people. Previously, these devices started as measure-specific nodes with limited memory and processing capacities, interconnected as a pervasive sensor network, and then turned to middleware integration in complex systems (Yang et al., 2010). Complex implementations (*e.g*., smart cities) also require the integration of physical artifacts (*e.g*., lights, buses), augmenting these artifacts to support people (Perera & Zaslavsky, 2014). Nowadays, AmI applications run in multiple environments (*e.g*., through a city), maintaining context-aware support close to users. Thus, ambient intelligence applications require enhanced governance, *i.e*., the capacity to control components for efficient service delivery within the system.

AmI systems extend service architecture styles to integrate context from devices and external systems, considering configurations, preferences, and the internal system (virtual context). A well-known architecture relies on a central server to manage all components (*e.g*., centralized governance in the cloud). Other implementations release the architecture deploying service aggregators such as gateways (or proxies), bringing capacities/governance near the devices. On the other hand, AmI implementations also provide self-governance adopting agent-based components following the autonomic computing paradigm. The purpose of this review is to analyze AmI service architectures and governance, structuring the variability of implementations based on conceptualization.

We have conducted a systematic mapping process to analyze and visualize a systemic situation in AmI systems with the following objectives:

1. To identify what the common service-based features used in AmI systems are;
2. To analyze what type of contexts are used and whether the applications consider the service itself as a context;
3. To review different architectures and how they manage the quality of service, governance, and security; and
4. To analyze research challenges in AmI service systems.

### Related work

Previous reviews have assessed specific SOA characteristics applied to AmI systems, identifying numerous related challenges. Truong & Dustdar (2009) have studied context-aware web service systems, envisaging the importance of SOA styles for Internet-based applications. The authors have identified the need for further support to interoperate between organizations, including security, context management, and quality. Stavropoulos, Vrakas & Vlahavas (2011) have analyzed the composition process and techniques, bringing the demand for quality, context-awareness, and managing user preferences. Knappmeyer et al. (2013) have reviewed the representation, management, reasoning, and evaluation strategies of context middleware approaches. The authors have identified challenges such as governance and the demand for context-aware service composition and deployment. The authors also envisage emerging middleware such as cloud computing and the required security and privacy. Perera & Zaslavsky (2014) have studied context-aware service systems from an Internet of Things (IoT) perspective. The authors identified challenges such as the adaptability of sensors, the governance and context discovery, security and privacy, and the way towards a sensing-as-a-service model inside the IoT. In this survey, we attempt to characterize these challenges.

### Rationale for the review

Ambient Intelligence has a significant number of service architectures and implementations. Querying research databases, we find projects that apply aspects of AmI and SOA. However, a small number conceptualizes the characteristics inside each approach or takes advantage of adopting complementary aspects such as defining service contracts for interoperability. For example, implementation efforts use web services without a registry, coding the services manually. On the other hand, a SOA-based implementation with service contracts provides a standard definition of services within the system. Thus, this review provides an overview of AmI systems adopting SOA characteristics. To the best of our knowledge, there are no systemic evaluation mapping AmI and SOA characteristics. This review presents a conceptual schema, organizing the core concepts to describe AmI and SOA aspects. It includes well-known definitions, principles, and structures of the research efforts. The review excludes subjective aspects such as visions, hypothetical scenarios, thoughts, and computational limitations. In addition, the preview reviews (related work) have identified challenges regarding only specific characteristics (*e.g*., improving context-awareness). Our goal is to identify new challenges and evolution with an integral view of SOA styles inside AmI systems.

### Intended audience and organization

This review focuses on the services dimension with a global overview of architectures to orchestrate ambient intelligent services, including cloud and intelligent agents providing ubiquitous services. We target this review for researchers and developers who require a conceptualization for new IoT application modeling and implementation. This review also explores the distributed service architectures evolving with 5G and new distributed computation models.

The following Section “AmI and SOA: Definitions and Related Technologies,” introduces the conceptual schema to analyze with characteristics of AmI and SOA styles. This schema considers existing implementations, discarding futuristic aspects. Based on our conceptual schema, Section “Review Methodology,” defines the review structure and details the method adopted for the study, including criteria for inclusion/exclusion. Section “Data Analysis: Systematic Map,” presents our analysis *per se* with the classification and mapping. Section “Results and Discussion,” presents the overall results and introduces the identified challenges. Finally, Section “Conclusions,” finishes the review with conclusions.

## Ami and soa: definitions and related technologies

Ambient Intelligence (AmI) systems integrate everyday objects and automate tasks in a transparent and pervasive multi-environment service provider. Nowadays, AmI systems adopt SOA to integrate/distribute computational services for a diversity of applications. AmI applications cover the intelligent management of context in houses and buildings, public and private places and businesses, leisure and tourism, digital spaces and assistants, and the Internet and Web of Things (Sadri, 2011). Also, AmI systems target e-health and personal health applications. Health systems include smart bio-signals sensing that feeds monitoring applications and affective and responsive systems aware of the body and environment, triggering actions to improve health conditions (Acampora et al., 2013; Sadek et al., 2018). This section describes AmI and SOA concepts to introduce an aggregate view for the survey, associating AmI and SOA approaches.

### Ambient intelligence (AmI)

Ambient intelligence is sensitive and sensible surroundings. AmI applications gather context using wireless or wired^1^ 1Wired gathering includes electrical, oil/gas control systems (*e.g*., SCADA (Supervisory Control and Data Acquisition) and DCS (Distributed Control Systems) facilitate and automatize sensing and human decision making) (Galloway & Hancke, 2013) technologies. For example, in ad-hoc/wireless sensor networks, distributed sensor nodes use Wi-Fi, ZigBee, Bluetooth, or Z-wave protocols to gather information from the environment or a monitored field (Rawat et al., 2013). Afterward, AmI applications apply artificial intelligence algorithms to adapt and provide support through interaction with the environment (Augusto, 2007; Cook, Augusto & Jakkula, 2009).

Augusto & Mccullagh (2007) define AmI as “A digital environment that proactively, but sensibly, supports people in their daily lives”. Sensible means a rational/reasonable system settled according to reason. Simultaneously, the system is sensitive to the needs, allowing interaction in a proactive and context-aware manner (Sadri, 2011). Being sensible entails recognizing the user (*e.g*., through learning and awareness of user’s preferences) and the capability of exhibiting empathy for their needs, desires, moods, the current situation, and social aspects (Augusto & Mccullagh, 2007). Nowadays, advances in technology provide the means for AmI systems to understand natural human interaction. They are responsive to commands (*e.g*., voice) and act based on user needs through pattern recognition (*e.g*., face, gesture) and behavior prediction (Dunne, Morris & Harper, 2021).

The fundamental element in AmI is context. Context is a digital representation of real conditions, considering the internal and external aspects of the system. Relevant to user and application interaction, context is any information used to characterize the situation of an entity (entity is a person, a place, an object, an application) (Dey, 2001), including the characteristics of the entity and application’s domain (Ponce, Roy & Abdulrazak, 2016). In autonomic computing, entities also represent the self as context, working with locally accessible information that includes their settings, operations, and semantics (Abdulrazak et al., 2010b). In an environment, the sensors allow gathering context, and the actuators enable the interaction. When the context is available, the system represents it through context management processes. The system then applies different data processing and reasoning techniques, providing ambient adaptation and digital support (Table 1). The general structure of an AmI system considers the following (Augusto & Mccullagh, 2007):

**Table 1:** Characterization of ambient intelligence systems.

Aspect	Uses	Approaches to implementation
Context lifecycle	Representation	Symbol table (Baldauf, Dustdar & Rosenberg, 2007), *e.g*., key-value Markup (Baldauf, Dustdar & Rosenberg, 2007), *e.g*., HTML, XML Logic-based (Baldauf, Dustdar & Rosenberg, 2007) Ontic-based (Roy, Abdulrazak & Belala, 2014) Graphical (Baldauf, Dustdar & Rosenberg, 2007), *e.g*., UML-based Object-oriented-based (Baldauf, Dustdar & Rosenberg, 2007) Ontology-based (Baldauf, Dustdar & Rosenberg, 2007) Graph-based (Kamienski et al., 2017)
	Management	Acquisition: query [the source]-based, event-driven, introduced by the user, *etc*. Preprocessing (process raw data) in a centralized, distributed, or hybrid scheme Inconsistency and resolution of conflicts through user interference, based on the quality of context, *etc*.
	Reasoning	Based on AI reasoning and learning techniques, *e.g*., description logic, situation calculus, dynamic belief networks
	Adaptation (context-awareness)	Context-based (Knappmeyer et al., 2013): query context on demand (synchronously) and adapt based on current context parameters, *e.g*., a mobile application that query GPS coordinates at lunchtime to notify nearby restaurants Context-aware (Knappmeyer et al., 2013): event-based (asynchronous) context diffusion and adapt based on events of interest, *e.g*., a mobile application registered to a restaurant, and receive a notification when the user is nearby and at lunchtime Situation-aware (Knappmeyer et al., 2013): higher-level aggregation of context. The adaptation considers historical context, current user activity, a selected plan, *e.g*., an application recommending a restaurant regarding past taken meals (time/place), and previous accompanying people Social-aware (Lukowicz, Pentland & Ferscha, 2012): a special case of situation-aware. The adaptation considers the synergy of entities, communities, and social dynamics, *e.g*., an application recommending a route based on traffic congestion and commuting patterns.
	Security	Based on information security principles, *e.g*., context-based access control
	Dissemination	Based on the ubiquity and middleware distribution approaches
Intelligence	Represent, reason, learn	User model, spatial & temporal reasoning, activity recognition, planning & decision-making. Multiple algorithms and models, *e.g*., probabilistic, propositional logic, fuzzy logic, AI Planning, natural language processing, deep learning.
Ubiquity	Interaction	Human-Computer: Unobtrusive interaction, *e.g*., haptic, User eXperience (UX) design, augmented reality, tangible (Ishii & Ullmer, 1997; Poslad, 2009)⁠ Computer-Computer: based on computer support, *e.g*., machine-to-machine, smart traffic, telemetry, multi-agent Things: system's extension with tagging or modeling, *e.g*., RFID, NFC, bio-chips, profile/shape-of-the-thing models (Poslad, 2009) Sensors/actuators, *e.g*., wireless sensor networks, global position systems, participatory sensing, SCADA
	Distribution	Based on network communication and distributed systems, *e.g*., peer-to-peer, publish/subscribe, client/server, mobile code
	Mobility	Distribution based on the capacity to move, *e.g*., m-health (mobile health system), pervasive
	Autonomy	Based on the autonomic computing approach, *e.g*., self-organization
	Reasoning	Based on intelligence and context awareness, *e.g*., semantic interoperability, smart-spaces

- Environment — the source and final supported entities (*e.g*., user, robot, another system).
- Sensors and actuators — people, systems, and devices producing context, gathered through sensors and receiving commands through actuators.
- Middleware — API, virtual machines, network devices, *etc*., for system distribution, gathering, processing, and actuating.
- Decision-making engine — reasoning with a knowledge repository for a proactive or reactive adaptation.
- Discovery and learning — To update the decision-making engine with experience over the whole system.

Context-awareness characterizes AmI systems. Schilit & Theimer (1994) introduced context-aware computing as—a capability of the applications to discover and react based on variation in the environment (Schilit & Theimer, 1994). Abowd, Dey & Brown (1999) extended the scope of awareness, highlighting the context’s significance, where information and services are relevant to the user’s task. Roy (2019) considered the scope of context-aware agents’ mission performing their micro assessments.

AmI aims to ensure a distributed, suitable, convenient, and precise service provision, avoiding overloading users. During the interaction in the system, context-awareness mechanisms provide intelligent management of the context (Augusto, 2007). Context-awareness applies to systems as advances are in artificial intelligence research (Cook, Augusto & Jakkula, 2009). Reasoning, Natural Language Processing (NLP), pattern recognition, prediction, and learning lead AmI systems towards a higher level of sensitivity and adaptability; and, as a result, to better support users (Zhang et al., 2011; Dunne, Morris & Harper, 2021). In ubiquitous scenarios, the awareness enables adaptation to different environments and provides distributed, pervasive and smart support (Augusto, 2007). Following, we describe the main aspects that highlight AmI systems (Augusto, 2007; Poslad, 2009; Truong & Dustdar, 2009; Cook, Augusto & Jakkula, 2009; Zhang et al., 2011; Christin et al., 2013; Dunne, Morris & Harper, 2021) (Table 1):

- Context-awareness, producing a reaction or pro-action based on the context gathered from the self-system and the environment; or integrating knowledge from the entities; the awareness interprets synchronous, asynchronous, situation-based, and social-based information changes.
- Intelligence, involving algorithmic support for decision-making and learning; the algorithms complement different aspects of the system.
- Ubiquity, distributing/integrating information from spread entities for providing an everywhere and an every-time provision; the entities extend the perception and interaction to the ambient, enabling the use of available devices, objects, and other entities.

Context-awareness is part of a context lifecycle where we find approaches to model, represent, process, and disseminate the context (Zhang et al., 2011). Ubiquity involves interaction design and distributed computing (*e.g*., ubiquitous or ambient computing). AmI relates human-computer interaction (HCI), smart sensor and actuator technologies, participatory sensing, embedded and machine-to-machine technologies, wired and wireless sensor networks, mobile networks, pervasive computing, cloud computing, and the IoT (Poslad, 2009; Zhou, 2012; Christin et al., 2013; Acampora et al., 2013) (Table 1).

Applications in AmI systems demand an architecture style, considering multidimensional design principles (*e.g*., interaction design, sensor/actuator management). We examine one dimension in this survey: the provision of service.

### Service-oriented architecture

Service-based systems have achieved a significant level of formalization and promote the strengthening of software design (Bianco et al., 2011; Nacer & Aissani, 2014) and have reached a broad deployment in business (Van-der-Aalst, 2013). Emerging technologies such as cloud computing (Rahimi et al., 2013) and the IoT (Xu, He & Li, 2014) also adopt service designs. SOA is the reference model for service-based systems. The SOA model applies the separation of concerns design paradigm and defines the service as the solution logic. A service abstracts functionality, hiding underlying details, executing actions as autonomous entities. SOA establishes a set of interrelated design principles^2^ 2Common service-orientation principles establish that services are reusable, share a formal contract, are loosely coupled, abstract underlying logic, are composable, are autonomous and are stateless (Erl, 2005). for the architecture (Erl, 2005; Bianco et al., 2011) and associates the following aspects:

- Interoperability, based on the standardization of interfaces, components’ inputs/format, and a formal definition of elements for sharing services (service contract). The formal definition includes an implicit or explicit semantic description. This aspect facilitates integration, adaptation, validation, federation; and supports security.
- Loose coupling, by designing self-contained services (*i.e*., with independent technologies and capabilities) with communication through messages, ideally agreed with a service interface description. This aspect facilitates the integration, adaptation, and it is the basis for building scalable architectures.
- Composability, by using reusable and compatible services to compose or aggregate functionality. This aspect augments the reactive or proactive capabilities for diverse scenarios.
- Reusable services, creating generic capabilities, and designing stateless services so that different consumers use the same services. It also involves mediators for services and mechanisms for discovery (find services to reuse).
- Discoverability, facilitating the reaching of services at design or runtime, maintaining a registry (or directory) of services for querying the required ones for future invoking.

Using SOA principles promotes the quality of service in the system. However, it leads to unbounded and unnecessary overhead processing (Bianco et al., 2011). Despite this, SOA provides a formal and well-designed framework for ambient service-based support systems and is the reference service model for this review.

A service is a well-defined capacity of an entity to perform a demand, being significant when another entity requires or utilizes it. Spohrer et al. (2007) define a service as “a kind of action, performance, or promise that is exchanged for value between provider and client”. The exchange between both entities—provider and client (or consumer)—requires standardization, *i.e*., using an interface contract, a shared syntax, and guaranteeing a service level agreement (SLA)^3^ 3SLA is a formal (contractual) agreement between a provider and a consumer. The aim is to define a consumer’s expectations.. The value represents the exchange that influences the level of satisfaction of the client/consumer (Kritikos et al., 2013).

The SOA infrastructure provides the means to interchange services between providers and consumers. The SOA model and principles are the guidelines for various service architectures (Papazoglou, 2003; Huhns & Singh, 2005; Bianco et al., 2011). We summarize the structure of SOA (Table 2), considering the four aspects: (1) service communication and integration, (2) application logic, (3) service registry and repository, and (4) monitoring and management. The traditional service-based systems adopt three architectural styles: (1) SOA-based, (2) Web services (WS), and (3) self-service systems (Spohrer et al., 2007) (Table 2). The WS and self-service follow the SOA model; however, the distinction lies in adopting Web protocols and autonomic computing (respectively). In general, SOA-based and WS architecture governance is implemented in a central component. Self-service governance is implemented on distributed components (*e.g*., agents) interacting based on policies, managing their behavior and relationship (Kephart & Chess, 2003), providing self-governance.

**Table 2:** Structure of SOA, WS, and self-service.

SOA (Papazoglou & Heuvel, 2007; Merson et al., 2011)⁠	XML Web-Services (WS), Semantic WS (Nacer & Aissani, 2014)⁠ and REST WS (Pautasso, 2014) ⁠	Self-Service (White, Hanson & Whalley, 2004)⁠
**Communication and integration:** With a broker or enterprise serial bus (ESB) as an intermediary between provider and consumer, that allows for a normalization of the interaction. It is not necessary for homogeneous environments.	Two ways: (1) Developing an interface; (2) using a communication/integration layer which either (a) results in a point-to-point communication or (b) involves an ESB. Language/protocol examples (♣ XML, ● Semantic, ♦ REST): Description: (♣) WS Description Language – WSDL; (♦) HTTP Verbs: GET, POST; (●) Resource Description Framework — RDF, Web Ontology Language (OWL), RDF Schema, OWL-S, WSDL-S, WS Modeling Ontology (WSMO) Communication: (♣) Simple Object Access Protocol — SOAP; (●) (♦) HTTP Message format: (♣) SOAP (represented with XML); (♦) XML, JSON, HTML, CSV; (●) XML triple, RDF, OWL, WS Modeling Language — WSML ontology	Broker: To facilitate the interaction, intermediary between provider and consumer
**Application logic:** Through an execution engine, *e.g*., a Business Process Engine. Also coded manually in the consumer/provider	Idem as service-oriented architecture (SOA)	Negotiator: To assist in complex decisions Aggregator: To combine services
**Service Registry and Repository:** To manage the directory, reaching, dependency, versioning, and federation capabilities	Through service registry services, *e.g*., application server service repository, universal description, discovery and integration protocol — UDDI, marketplace	Registry: To manage the directory and to reach services
**Monitoring and Management**: To monitor services and maintain the quality of service	Implemented in the application server	Sentinel: To monitor services

### Services provided by ambient intelligence systems

Access to efficient services characterizes SOA systems. Efficiency is a salient attribute for the service consumer who requires a quality of service from the provider. Following, we identified the SOA features applied to AmI (Sheth, 1999; Kephart & Chess, 2003; Bianco et al., 2011; Stavropoulos, Vrakas & Vlahavas, 2011; Knappmeyer et al., 2013; Raychoudhury et al., 2013) which are the main aspects adopted for the survey (Table 3):

**Table 3:** Characteristics of AmI service-based systems.

Structure	Characteristic	Approaches to implementation
Communication and integration	Interoperability	Syntactic: Includes communication and integration using standards such as HTML, XML, SOAP Semantic: Communicates meaning. The basis is shared conceptualization and integration of a domain⁠
	Middleware distribution	Central Server: An application server(s) is responsible for the distribution in a server-client approach. Can include support from distributed components such as proxies or gateways Enterprise Serial Bus: Intermediary that allows the normalization of the distribution Peer-to-Peer: Each element acts as both a server and a client Autonomous Agents: The elements manage themselves and distribute in different ways (*e.g*., peer-to-peer)
Application Logic	Composition/Aggregation	AI Planning (algorithms): Based on a goal, connects the components Capabilities Matching: Match expressions (*e.g*., rules, script) with the entries of the registry or policies Agent Matching: With context-based negotiations based on rules inside the agent with semantic matching
Registry/Repository	Registry	Canonical Expression: Static service contract ● Use of an Intermediary (*e.g*., broker) Runtime registration: Dynamic registration at runtime ● No Registry: Coded manually
	Discovery	Query: Search inside the registry ● Subscriber: Publish/subscribe paradigm Direct Access: Pre-defined/coded manually ● Lookup: By broadcast/algorithm/semantic matching
Monitoring and management tools	Management	Admin Service: A service is responsible for coordinating the system. Can be an Admin Agent Self-Adaptable Governance: Self-management (self-config, self-optimization, self-healing, self-protection)
	Monitoring	Supervisor Service: A service monitors the system. Can be a supervisor agent (or Sentinel) Self-Adaptable Monitoring: Based on self-management aspects
	Quality of Service (QoS)	Service Performance: Considers the quality of service *per se*, *e.g*., QoS rate, availability of service Service Resources: Considers physical and logical resources, *e.g*., network, CPU, location, activity zone Service Context: Considers rules and functional policies to define the Quality of Context (QoC)

- In communication and integration, attributes to exchange context between entities and services, involving distribution and interoperability.
- In application logic, approaches for the composition and the aggregation of services, including algorithms to the couple, reuse, and organization of services.
- In monitoring and management, mechanisms for governance: managing, monitoring, and maintaining available and efficient services.
- In registry and repository, attributes for building visible services and approaches to use services before and after deployment.

**Figure 1:**
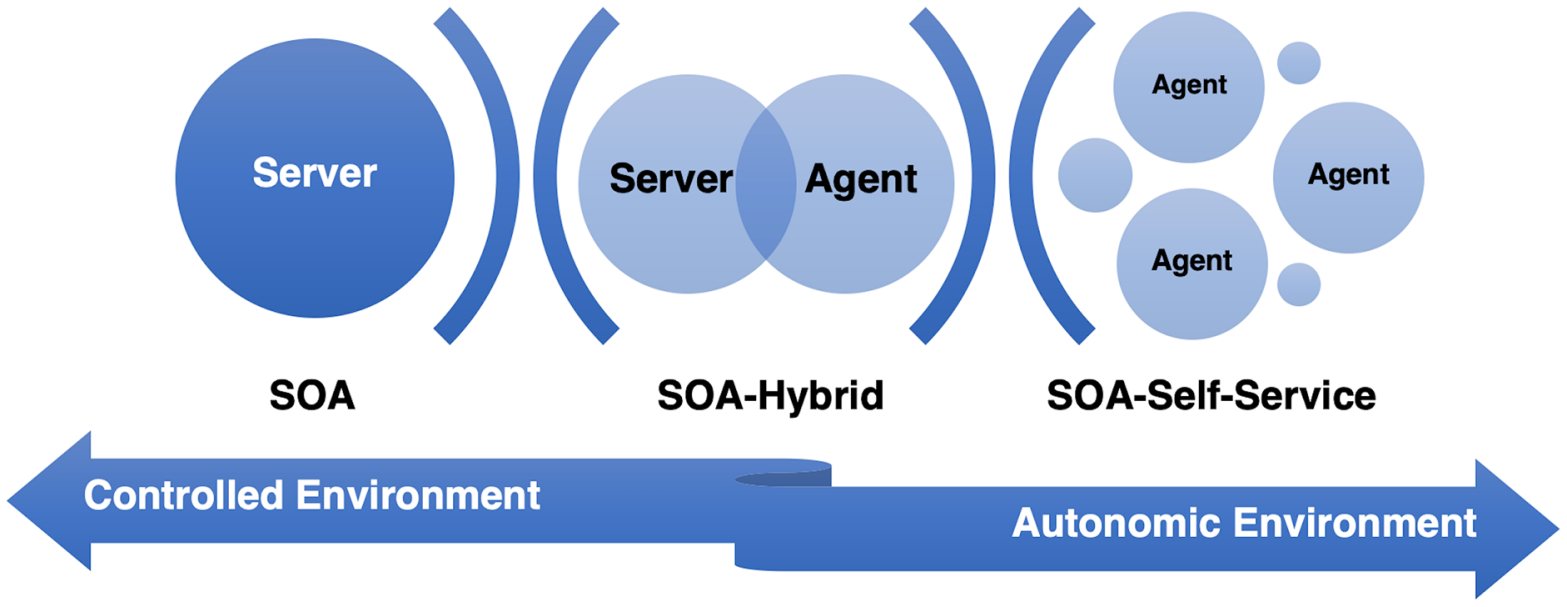
Ambient intelligence service architecture styles.

Ambient intelligence applications demand service governance, *i.e*., capacity to control components for providing efficient services. AmI applications also require autonomy in environments, such as in smart cities, independent living, and e-health. Service-based AmI systems provide governance following SOA principles and patterns. We define three types of architectural styles for AmI service systems: SOA-based, self-service, and hybrid: (1) SOA-based are when governance is mainly on a server (even though having distributed components), enabling a controlled environment, *e.g*., a central server, cloud, (2) Self-service are when agents provide self-governance, enabling an autonomic provision, and (3) hybrid when the governance involves both, *e.g*., with agents, fog computing (Fig. 1).

We have presented in this section a conceptual schema that characterizes AmI (Table 1) and service-based AmI systems (Table 3). In this survey, we intend to find the synergy between AmI systems and SOA. Therefore, we selected applications to assess interrelated AmI-SOA characteristics. In the following section, we describe our method for the analysis.

## Review methodology

**Figure 2:**
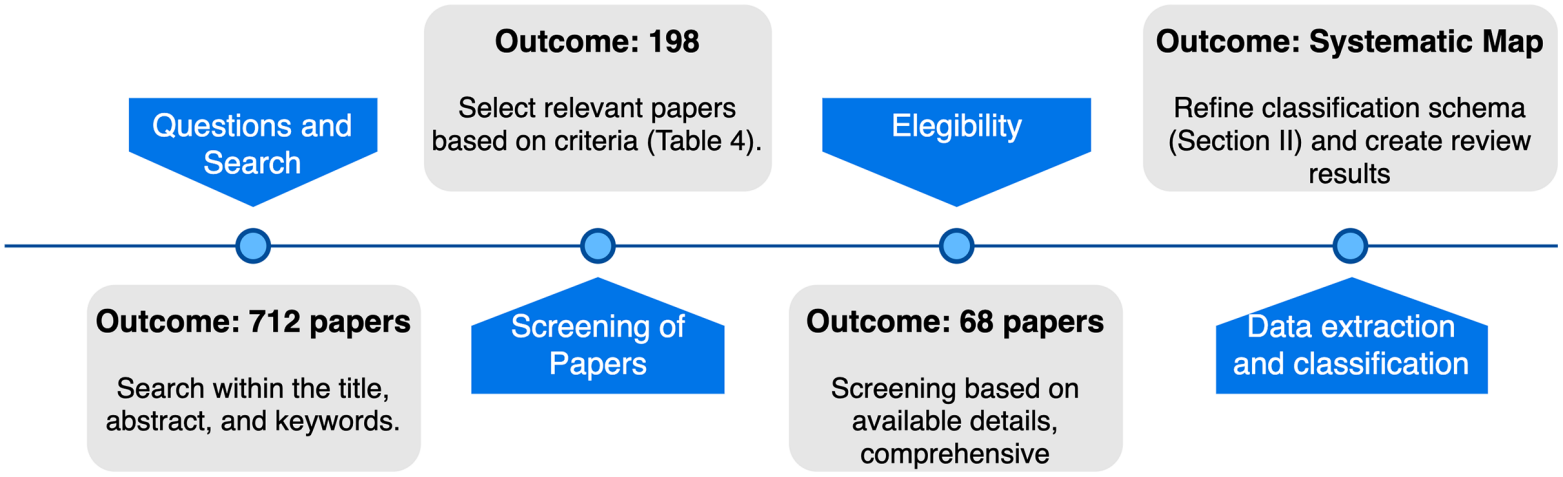
Review method.

We adopted the systematic mapping process—a literature review methodology to build a categorization scheme that structures the types of research reports and results in published papers (Petersen et al., 2008). The adopted process relies on the definition of research questions to search relevant papers to extract data. The outcome of the process is a systematic map with the frequencies of publications based on a classification scheme. Our method (Fig. 2) begins with determining research questions and continues searching for published papers in research databases. After, we screened the papers based on our inclusion criteria, selecting 68 relevant research projects for analysis (see Table 4 for details). Then, we defined the classification scheme, which also refined our conceptualization (Section “AmI and SOA: Definitions and Related Technologies”). Finally, we evaluated the selected papers, extracting data and creating a mapping of studies.

**Table 4:** Screening of papers.

Consideration	Inclusion	Observations
Publication year	2001 to 2021	The final range is 2006 to 2021
Relationship with the subject	AmI AND SOA characteristics	Based on the conceptualization
Research type (Petersen et al., 2008)⁠	Proposal of solutions and philosophical papers	**Proposal:** a novel, extended technique. It may include proofs-of-concept. **Philosophical:** model/framework.
Available details, comprehensive	Description with an argumentation	Based on the details (components, tools, algorithms, *etc*.)
Type of Document	Proceedings, journals	IEEE, ACM, SCOPUS, Web of Science

### Research questions

The following are our research questions (RQ) and their aim (Table 5):

**Table 5:** Classification scheme and contribution to the review.

Facet	Attribute	Objective	Aim*	
Ambient intelligence	Domain	To identify the variety of uses	2, 4	
	Awareness	To identify the awareness for adaptation	2,4	
	Mobility	To identify if components are in mobile devices	1, 3, 4	
	Service-as-a-Context	To determine the trend of considering the service in context-aware processing	2, 4	
	Agent-Based	To determine the trend of using agents	1, 3, 4	
Service styles	Architecture	To classify as SOA, self-service, or hybrid	1, 3, 4	
	Registry/Repository	To identify applied techniques for registering and discovering	1, 3, 4	
	Distribution	To identify applied techniques	1, 3, 4	
	Composition	To identify applied techniques	1, 3, 4	
	Interoperability	To identify the use of semantic communication	1, 3, 4	
	Management	To identify the applied management techniques	1, 3, 4	
	Monitoring	To identify the applied monitoring techniques	1, 3, 4	
	QoS	To identify the use of quality attributes	1, 3, 4	
Research/Global	Publication	To identify the research over time	4	
	Security	To identify the applied security mechanisms	3, 4	
	Research Type	To classify as solution proposal or framework	4	

**Note:**

* Aim: 1: SOA features on AmI; 2: Variety of context; 3: Infrastructure; 4: Identifying challenges.

1. **Aim: Identify SOA features on AmI.** RQ: What service characteristics are implemented in ambient intelligence? To identify what the common service-based features used in AmI systems are.
2. **Aim: Analyze the variety of contexts.** RQ: What is the variety of contexts? To analyze the applications’ context types and whether they consider the service itself as a context.
3. **Aim: Review architecture styles.** RQ: What are the implemented architectures in ambient service systems? To review existing architectures and how they manage the quality of service, governance, and security.
4. **Aim: Identify and analyze challenges.** RQ: What are the challenges? To analyze further research challenges in AmI service systems.

### Search strategy

We searched using the title, abstract, and keywords with different syntax is depending on the target research database. The generic search string is the following:

(ubiquitous OR pervasive OR context-aware* OR “Internet of Things” OR IoT) AND (“ambient intelligence” OR “smart environment” OR “smart space” OR “smart habitat” OR “smart city”) AND (“service-oriented” OR SOA OR “web service” OR (autonomic AND service) OR “microservice”).

We conducted searches on the following research databases: IEEE Xplore, ACM Digital Library, Scopus, and Web of Science.

### Inclusion/exclusion criteria

After conducting searches in research databases, we obtained 712 papers. Subsequently, we examined and selected suitable papers based on the following criteria (Table 4): The year of publication, the relationship with the subject, research type, comprehensibility, and the type of document (*i.e*., proceedings and journal papers). The range of the year of publication started in 2001, based on the growth of research in ubiquitous and pervasive computing (Zhao & Wang, 2010). The relation with the subject depends on the fulfillment of the conceptualization. The type of research characterizes the contribution of the paper (Wieringa et al., 2005). The survey only considers papers that propose a solution with proof-of-concept and philosophical papers that propose a model/framework. We discarded papers that evaluate a system, opinion papers, and papers describing personal experiences. We evaluated papers with understandable information, complementing the analysis with their related works and references.

### Data extraction and classification

After a first screening, we selected 198 papers for a detailed screening. After a second screening of the papers, we analyzed the information of 68 papers corresponding to 48 research projects. Nine projects include progressive works with two or more papers (*e.g*., Abdulrazak et al. with two papers: Abdulrazak et al., 2011; Roy, Abdulrazak & Belala, 2011). Subsequently, we defined the final classification scheme using the selected papers and the conceptualization (Section “AmI and SOA: Definition and Related Technologies”).

Table 5 lists the classification facets and their contribution to the research questions of the survey. In general, the papers focus on service architecture, and we could match most of the service-based characteristics (Table 3) in the Service facet. Conversely, related to the AmI characteristics (Table 1), the papers describe mostly the awareness (for adaptation) and ubiquity (*i.e*., distribution, mobility, autonomy) included in the AmI facet. We also include aspects in the AmI facet regarding the research questions:

- We include the target domain to identify a variety of uses. It follows Sadri (2011), where the author studied AmI projects by domain. The domains include, among others, applications for smart home, assisted living, healthcare, business and commerce, leisure and tourism, human-inspired (integrating emotional user data), as well as projects for general data management (*e.g*., sensor networks) and AI techniques (*e.g*., self-organized robot control).
- We include whether the service is considered part of the context (service-as-a-context) and its inclusion in context-awareness (*e.g*., improving the quality of service).
- We consider whether the projects include agent technology due to the agent-based software approach’s contribution to AmI systems (Cook, Augusto & Jakkula, 2009).

We also introduce the research/global facet that comprises the publication year, whether the project considers security aspects and the research type. The research type follows the classification of the systematic mapping process presented in Petersen et al. (2008). Each project’s category derives from screening papers and identifying whether the project is a solution proposal (*i.e*., application for a particular domain) or a framework/model (*i.e*., general design).

The final step is creating a systematic map, being the basis for further analysis. In the following sections, we present the systematic map and discuss key findings.

## Data analysis: systematic map

We have analyzed each research effort based on the classification scheme (presented in the Section “Definition and Related Technologies”). We used different tools to group information during the analysis, build mind maps, and create lists and graphs for each facet (*i.e*., service, AmI, research/global). In the mapping process, we extracted detailed data to the extent that the information exists in the papers, including information from tables and figures when necessary. Tables 6 and 7 list the final categorization of the data extracted from the projects.

**Table 6:** Categorization in ambient intelligence and research and global facets.

	Year	Research project	Research type	Domain	Awareness	SaaC*	Agent-Based*	Mobility*	Security*
**Centralized**	2007	Baresi, Guinea & Pasquale (2007)	Sol. Proposal	Business–BPM	Situation-aware	✓	✗	✗	✗
	2007	Yin et al. (2007)	Framework	General–smart space	Context-aware	✓	✗	✗	✗
	2008	Athanasopoulos et al. (2008)	Framework	General–AmI	Situation-aware	✗	✗	✗	✗
	2009	Silva, Mouttham & Saddik (2009)	Sol. Proposal	Healthcare–prescriptions	Context-based	✗	✗	✓	✗
	2010	Prehofer & van Gurp (2010)	Framework	General–smart space	Context-based	✗	✗	✗	✓
	2010	Gouin-Vallerand et al. (2010)	Framework	General–smart space	Context-aware	✓	✗	✗	✓
	2010	Ahn & Nah (2010)	Framework	General–AmI	Context-aware	✓	✗	✓	✗
	2011	Paganelli & Giuli (2011)	Sol. Proposal	Healthcare	Situation-aware	✗	✗	?	✗
	2013	Yusro et al. (2013)	Sol. Proposal	Assisted Living	Context-aware	✗	✗	✓	✗
	2013	Albreshne, Lahcen & Pasquier (2013)	Sol. Proposal	Smart home	Context-based	✓	✗	✗	✗
	2013	Hasswa & Hassanein (2013)	Sol. Proposal	Emotional	Situation-aware	✗	✗	✓	✓
	2015	Yachir et al. (2015)	Framework	General–AmI + IoT	Context-aware	✗	✗	✗	✗
	2016	Triboan, Chen & Chen (2016)	Sol. Proposal	Assisted Living	Context-based	✗	✗	✓	✗
	2017	Gonzalez-Usach et al. (2017)	Sol. Proposal	Assisted Living	Context-based	✗	✗	✗	✓
**Distribution**	2011	Smirnov, Levashova & Shilov (2011)	Sol. Proposal	Institutions–emergency	Situation-aware	✓	✗	✓	✗
	2012	Amoretti (2012)	Framework	Data–AmI	Situation-aware	✓	✗	✓	✓
	2014	Pan et al. (2014)	Framework	General–AmI	Situation-aware	✗	✗	✗	✗
	2014	Nosović, Peters & Bruegge (2014)	Framework	General-AmI	Context-aware	✗	✗	✗	✓
	2017	Prado, Ortiz & Boubeta-Puig (2017)	Framework	General-IoT	Situation-aware	✓	✗	✗	✗
**Distributed**	2009	Kawashima et al. (2009)	Framework	General–smart space	Context-aware	✗	✗	✓	✗
	2009	Bernardos, Tarrío & Casar (2009)	Framework	General–AmI	Situation-aware	✗	✗	✓	✗
	2010	Reetz, Tonjes & Baker (2010)	Framework	Data management–WSN	Situation-aware	✗	✗	✗	✗
	2011	Roggen et al. (2011)	Framework	General–AmI	Context-aware	✓	✗	✓	✗
	2013	Degeler et al. (2013)	Sol. Proposal	Smart Building	Context-aware	✗	✗	✗	✗
	2013	Stavropoulos et al. (2013)	Framework	General–AmI	Context-based	✗	✗	✓	✗
	2013	Buzeto et al. (2013)	Sol. Proposal	Emotional–games	Context-aware	✗	✗	✗	✗
	2014	Forkan, Khalil & Tari (2014)	Sol. Proposal	Assisted Living	Situation-aware	✗	✗	✗	✗
	2014	Dar et al. (2015)	Framework	Business–BPM + IoT	Context-aware	✗	✗	✓	✗
	2016	Fysarakis et al. (2016)	Framework	General–AmI	Context-based	✗	✗	✗	✓
	2017	Lee et al. (2017)	Framework	General-IoT	Context-aware	✗	✗	✗	✗
	2019	Malik & Kim (2019)	Framework	General-WSN/IoT	Context-based	✗	✗	✗	✗
	2020	Pitatzis et al. (2020)	Sol. Proposal	General–AmI + IoT	Context-aware	✗	✗	✗	✗
	2020	Javed et al. (2020)	Framework	Smart cities	Context-based	✗	✗	✗	✓
	2021	Gooder et al. (2021)	Sol. Proposal	General-smart environments	Context-aware	✓	✗	✗	✓
**Intelligent agents**	2006	Soldatos et al. (2006)	Framework	General–smart space	Situation-aware	✗	✓	✗	✗
	2009	Miyata, Morikawa & Ishida (2009)	Sol. Proposal	Institutions–learning	Context-aware	✓	✓	✓	✗
	2012	Bhuvaneshwari & Sujatha (2012)	Sol. Proposal	Institutions–Traffic	Context-aware	✗	✓	✗	✗
	2012	Familiar, Martínez & López (2012)	Framework	Data–wireless, WSN	Context-aware	✗	✓	✗	✓
	2013	Tapia et al. (2013)	Framework	Data–WSN	Context-aware	✓	✓	✓	✓
	2015	Ferilli et al. (2015)	Framework	General–AmI	Situation-aware	✗	✓	✗	✗
	2016	Fysarakis et al. (2015)	Framework	General–AmI	Situation-aware	✓	✓	✗	✓
	2017	Mohamed et al. (2017)	Framework	Smart cities	Context-based	✓	✓	✗	✓
**Autonomic**	2006	Kim et al. (2006)	Sol. Proposal	Data–robot control	Situation-aware	✓	✓	✓	✗
	2010	Penserini et al. (2010)	Model	General–AmI	Context-based	✓	✓	?	?
	2011	Chun et al. (2011)	Framework	General–smart space	Situation-aware	✓	✓	✓	✗
	2011	Acampora, Loia & Vitiello (2011)	Sol. Proposal	Emotional	Situation-aware	✗	✓	✗	?
	2011	Abdulrazak et al. (2011)	Model	General–smart space	Situation-aware	✗	✓	✓	✗
	2018	Ruta et al. (2018)	Framework	Smart home/building	Situation-aware	✓	✓	✓	✗

**Note:**

* ✓ = Considered; ✗ = Not considered; ? = Undefined; SaaC = Service-as-a-Context.

**Table 7:** Categorization in service facet.

	Year	Research project	Style*	Registry*	Discovery*	Middleware*	Composition*	Interoperability*	Management*	Monitoring*	QoS*
**Centralized**	2007	Baresi, Guinea & Pasquale (2007)	SOA	Canonical	Query	Central server	Capabilities	Syntactic	✓	✓	✓
	2007	Yin et al. (2007)	SOA	Canonical	Query	Central server	Capabilities	Syntactic	✓	✓	✓
	2008	Athanasopoulos et al. (2008)	SOA	Runtime	Query	Central server	Capabilities	Semantic	✗	✗	✗
	2009	Silva, Mouttham & Saddik (2009)	SOA	✗	Direct	Central server	✗	Syntactic	✗	✗	✗
	2010	Prehofer & van Gurp (2010)	SOA	Runtime	Query	Central server	Capabilities	Syntactic	✗	✗	✗
	2010	Gouin-Vallerand et al. (2010)	SOA	Runtime	Query	Central server	✗	Semantic	✗	✗	✓
	2010	Ahn & Nah (2010)	SOA	Canonical	Query	Central server	✗	Syntactic	✓	✗	✗
	2011	Paganelli & Giuli (2011)	SOA	✗	Direct	Central server	✗	Semantic	✗	✗	✗
	2013	Yusro et al. (2013)	SOA	✗	Direct	Central server	✗	Syntactic	✗	✗	✗
	2013	Albreshne, Lahcen & Pasquier (2013)	SOA	Canonical	Query	Central server	Capabilities	Semantic	✓	✓	✗
	2013	Hasswa & Hassanein (2013)	SOA	Canonical	Subscriber	Central server	✗	Sem/Syn	✓	✓	✓
	2015	Yachir et al. (2015)	SOA	Runtime	Lookup	Central server	Cap+plan	Syntactic	✗	✗	✓
	2016	Triboan, Chen & Chen (2016)	SOA	Canonical	Query	Central server	Capabilities	Semantic	✗	✗	✗
	2017	Gonzalez-Usach et al. (2017)	SOA	Canonical	Query	Central server	Capabilities	Sem/Syn	✓	✗	✗
**Distribution**	2011	Smirnov, Levashova & Shilov (2011)	SOA	Canonical	Query	Central+agents	✗	Semantic	✓	✓	✗
	2012	Amoretti (2012)	Self	Runtime	Lookup	P2P	Planning	Semantic	✓	✓	✓
	2014	Pan et al. (2014)	SOA	Runtime	Query	ESB	Cap+plan	Semantic	✓	✓	✓
	2014	Nosović, Peters & Bruegge (2014)	SOA	Runtime	Query	ESB	Capabilities	Syntactic	✗	✗	✗
	2017	Prado, Ortiz & Boubeta-Puig (2017)	SOA	Runtime	Que+Subs	ESB	Capabilities	Syntactic	✓	✗	✗
**Distributed**	2009	Kawashima et al. (2009)	SOA	✗	Lookup	Central+gateways	✗	Syntactic	✓	✓	✗
	2009	Bernardos, Tarrío & Casar (2009)	SOA	Canonical	Que+Subs	Central+gateways	Capabilities	Syntactic	✓	✗	✓
	2010	Reetz, Tonjes & Baker (2010)	SOA	Runtime	Subscriber	Central+gateways	✗	Syntactic	✗	✓	✗
	2011	Roggen et al. (2011)	SOA	Canonical	Lookup	Central+gateways	Capabilities	Syntactic	✓	✓	✓
	2013	Degeler et al. (2013)	SOA	Canonical	Subscriber	Central+gateways	Cap+plan	Syntactic	✓	✓	✗
	2013	Stavropoulos et al. (2013)	SOA	Canonical	Query	Central+gateways	✗	Syntactic	✗	✓	✗
	2013	Buzeto et al. (2013)	SOA	Runtime	Subscriber	Central+gateways	Capabilities	Semantic	✗	✗	✗
	2014	Forkan, Khalil & Tari (2014)	SOA	Canonical	Query	Central+gateways	Capabilities	Syntactic	✓	✗	✗
	2014	Dar et al. (2015)	SOA	Runtime	Que+Subs	Central+P2P	Capabilities	Syntactic	✓	✓	✓
	2016	Fysarakis et al. (2016)	SOA	Canonical	Lup+Subs	Central+gateways	Capabilities	Syntactic	✗	✗	✗
	2017	Lee et al. (2017)	SOA	Canonical	Query	Central+gateways	Capabilities	Syntactic	✗	✓	✗
	2019	Malik & Kim (2019)	SOA	Canonical	Query	Central+gateways	Capabilities	Syntactic	✓	✗	✗
	2020	Pitatzis et al. (2020)	SOA	Canonical	Query	Gateway+mservices	Capabilities	Syntactic	✓	✓	✓
	2020	Javed et al. (2020)	SOA	Canonical	Query	Central+ gateways	Capabilities	Sem/Syn	✓	✗	✗
	2021	Gooder et al. (2021)	SOA	Runtime	Que+Subs	Microservices	Capabilities	Syntactic	✓	✓	✓
**With agents**	2006	Soldatos et al. (2006)	Hybrid	Runtime	Query	Central+agents	✗	Semantic	✓	✓	✓
	2009	Miyata, Morikawa & Ishida (2009)	SOA	Canonical	Query	Central+agents	✗	Syntactic	✗	✗	✓
	2012	Bhuvaneshwari & Sujatha (2012)	SOA	Runtime	Subscriber	ESB+agents	Cap+plan	Semantic	✗	✗	✗
	2012	Familiar, Martínez & López (2012)	SOA	Canonical	Subscriber	Central+agents	Capabilities	Syntactic	✓	✗	✓
	2013	Tapia et al. (2013)	SOA	Canonical	Query	Central+agents	✗	Syntactic	✓	✓	✓
	2015	Ferilli et al. (2015)	Hybrid	Canonical	Query	Agents	Capabilities	Semantic	✓	✓	✗
	2016	Fysarakis et al. (2015)	Hybrid	Runtime	Query	Central+agents	Capabilities	Syntactic	✓	✓	✓
	2017	Mohamed et al. (2017)	Hybrid	Runtime	Lookup	Central+agents	Capabilities	Syntactic	✓	✓	✓
**Autonomic**	2006	Kim et al. (2006)	Self	Runtime	Query	Agents	Agent match	Syntactic	✓	✓	✗
	2010	Penserini et al. (2010)	Hybrid	?	?	Agents	Agent match	Undefined	✓	✗	✗
	2011	Chun et al. (2011)	Self	✗	Direct	Agents	Agent match	Syntactic	✓	✓	✗
	2011	Acampora, Loia & Vitiello (2011)	Hybrid	?	?	Agents	?	Syntactic	?	?	?
	2011	Abdulrazak et al. (2011)	Self	Canonical	Lookup	Agents	Agent match	Semantic	✓	✓	✓
	2018	Ruta et al. (2018)	Self	Canonical	Lookup	Agents	Agent match	Semantic	✓	✓	✓

**Note:**

* ✓ = Considered; ✗ = Not Considered; ? = Undefined; Que = Query; Subs = Subscriber; Lup = Lookup; Self = Self-service; Cap = Capabilities; Plan = AI planning.

In this section, we start with a description of each selected research project. Then, we present the overall results and illustrate the relevant aspects of each facet.

### Service governance in ambient intelligence systems

We classify the projects into five groups related to the governance design within the architecture style (*i.e*., SOA-based, self-service and hybrid):

- centralized governance, a broadly implemented approach with a controlled architecture;
- centralized governance with distribution, centralized but adding components for the distribution of governance;
- distributed governance, with service aggregators such as gateways (or proxies), bringing capacities/governance near the devices;
- governance including agents, *i.e*., components to spread the governance; and
- self-governance: Governance in autonomic/autonomous architectures.

In this section, we present our classification with a summary of the main aspects of the selected projects, describing the research’s objective, the architecture components, how they integrate into the context, and the relationship between context and the provided service. The description avoids technologies or specific implementations (unless essential to the description) to maintain a technology-independent mapping. As a rule, we identify each research effort with a “first author et al.” format. For the projects that include more than one research effort, we identify them using the latest paper.

#### Centralized governance

Service governance with centralized components is the traditional design strategy. Diverse software architects use an SOA-based framework to implement SOA compliance. Others apply SOA characteristics as needed, use Web Services and other technologies such as ontologies to provide service or interoperability meaning.

#### Centralized governance with a SOA-based suit/framework

Using SOA suits is an approach to include service orientation because they apply SOA compliance in their design. In addition, SOA-based suits provide centralized components to manage more services and many service compositions, use various communication protocols, and process multiple accesses/messages.

Baresi, Guinea & Pasquale (2007) have introduced the Dynamo framework to manage self-healing services. The framework is an aspect-oriented extended version of the ActiveBPEL process orchestration engine, including business rules (with the JBoss Rule Engine). In addition to business process management (BPM), the framework includes a monitor and a recovery manager. Based on rules, the engine performs monitoring with data collection and data analysis, considering internal and external variables and historical context such as performance deterioration in the past. This information is combined with a self-healing rule-based recovery strategy.

Yin et al. (2007) have presented an SOA and tool for service composition in smart spaces. The architecture comprises development and runtime environments. The development environment includes a GUI for the design of services and applications. The runtime environment contains three layers: the OSGi platform, middleware, and application layers. The OSGi platform provides SOA capabilities. The middleware enables OSGi service registration and discovery regarding user behavior and service capability attributes (*e.g*., QoS, context information). Finally, the application layer contains a category-based division of applications, composite services, and atomic services to facilitate reaction in need for alternative actions.

Gouin-Vallerand et al. (2010) have presented a context-aware SOA framework for smart spaces. Context-aware reasoning infers a device capability quotient (DCQ) to determine the quality of context produced by the devices and, as a result, the most suitable environment device to use. The framework adopts the OSGi platform SOA capabilities (Gouin-Vallerand et al., 2009, 2010). In addition, it implements communication and security protocols, an administration tool (UI), an environment manager (coordinator for deployment, discovery, gathering, and reasoning), and a device request manager. The framework also includes an ontology handler. Finally, it manages a smart space meta-ontology representing the being, environment, and dynamic tasks (Abdulrazak et al., 2010a).

Using SOA-based frameworks introduces components with non-required characteristics. Thus, diverse solutions implement SOA characteristics as needed. For example, Yachir et al. (2015) have presented FASEM – an event-aware framework for ambient intelligence. It introduces a Bayesian learning mechanism for QoS estimation, services invocation, and other processes. The architecture comprises five modules: Service discovery, service classification, user task specification, events handling, and service selection. The service discovery that is subscribed to service providers (*i.e*., directories) receives and registers services. The service classification creates classes of services based on a global ontology. The task specification enables users to create an ontology-based specification of tasks and requirements, *e.g*., event rules. The event handling subscribes to the events (defined in event rules) and triggers ambient services. Finally, the service selection invokes the triggered events from service providers.

#### Centralized governance with web services

Web Services (WS) is a well-known implementation approach because of its SOA compliance, standardization, and interoperability support. Athanasopoulos et al. (2008) presented an SOA framework for context management in AmI systems. The framework enhances the capabilities of a base middleware for mobile WS (Issarny, Sacchetti & Tartanoglu, 2005), facilitating the dynamic integration of context through additional services for context sources discovery, context aggregators, and context interpreters. The context discovery supports presence in the environment, as well as discovers and registers available WS interfaces. The context aggregator allows for defining relations, using custom rules for context gathering and processing, and producing relevant context information. The context interpreter is a context SQL assembler.

Silva, Mouttham & Saddik (2009) have handled the medicine prescription management problem for aging people in AmI. Implementation includes a centralized server supporting the scheduling and history of intakes. The server implements WS accessed by mobile clients. The architecture consists of an event manager and a personal health record (PHR) interface. The event manager handles intake feedback events and proactive alerts sent to physicians and relatives when the intake interrupts. The PHR service interface acts as a loose coupling proxy to external PHR information and prescription information.

Prehofer & van Gurp (2010) have presented a REST-based framework for smart spaces, including functionalities for finding and securing relevant local resources based on context information from devices and users. The framework consists of a web platform, middleware services, and web portal-based applications. The framework includes service registration and discovery through a search engine to support resource localization. The framework also provides security services and context services. Security services guarantee access to resources using authentication and authorization. Context services interface relevant resources to users.

Yusro et al. (2013) have presented the smart environment explorer stick (SEES)—a smart cane for people with visual impairment. SEES integrates data from a camera, GPS, wheel encoder, ultrasound, compass, and accelerometer, producing context to analyze aspects such as color, obstacles, route, surface roughness. SEES includes algorithms for image processing of traffic lights, tracking, location, alerts, *etc*. SEES consists of a global server (iSEE) and two subsystems: SEE-stick and SEE-phone. iSEE provides WS, such as localization and remote monitoring. The SEE-stick and SEE-phone are interconnected through WiFi, increasing the phone’s sensing/actuating capabilities with an augmented perception provided by the stick.

#### Centralized governance with ontologies

Nowadays, SOA frameworks also regard knowledge representation to improve governance because it enables maintaining exact service/context meaning. Diverse projects have considered ontologies to represent system components, to facilitate interoperability or both.

Ahn & Nah (2010) have presented a WS context-aware framework to allow users to use services anytime and anywhere. Registration and matching algorithms utilize a service category ontology that classifies service types (*e.g*., shopping, transport). The framework includes three components: service provider, service requester, and service broker. The service provider handles context integration from sensors (processing location data) and the service registry. The service requester manages the context-aware client. The service broker implements a service finder based on a matchmaking pattern, matching the requested category of service, location, and available communication protocols (*e.g*., ZigBee).

Paganelli & Giuli (2011) presented a context-aware SOA platform for home and continuous care support. The platform adopts the Web and semantic services, including three components: a multichannel healthcare services manager, a central and patient context manager, and a wireless sensor system. The multichannel service manager maintains an ontology-based assistance model and services, including patient records, profiles, and alerts. Both the central and patient context managers provide back-end services for processing context changes and deliver continuous patient support at a central care center or patient’s home. Finally, the wireless sensor system collects the patient’s biomedical data (*e.g*., heart rate) and environmental data.

Albreshne, Lahcen & Pasquier (2013) have presented an approach for modeling, discovering, orchestrating, and executing services for smart residential environments (SRE). It incorporates an ontology to describe the SRE and capabilities of devices and a domain-specific language (DSL) to design and control the environment. The framework comprises templates’ repository, process generator, execution environment, client user interface (CUI), and execution engine. The templates’ repository stores the templates defined by a process template tool using both the ontology and DSL. The process generator interprets a template and generates an executable. The execution environment handles SRE services. The CUI allows the definition of user preferences. The execution engine is the process runtime environment.

Hasswa & Hassanein (2013, 2011) have presented a smart social space framework to enhance users’ experience by providing adaptable multimedia services. It implements an ontology representation of the user’s profile, preferences, and services. The framework includes services to manage feeds and geo-feeds, location and presence, and user profiles from social networks. The architecture comprises communication services, an application server (interact and coordinate processes), presence and a policy server (propagates changes to subscribed users), a location awareness server (manages geographical information), a home subscriber server (provides persistence), client-user agent (mobile application), a social network manager, and a system manager.

Triboan, Chen & Chen (2016) have presented a REST-based solution for AAL. The architecture includes three components: WS platform, triplestore, and Android application. WS platform consists of the following layers: (a) smart WS API (exposes services), (b) façade (abstraction of complex and CRUD operations), (c) repository (knowledge base), (d) domain (data mapping among layers), and (e) utility (low-level processes, *e.g*., communication, ontology management). The triplestore layer includes a database to store triple datasets for semantic interoperability. The Android application provides the primary system interface.

Gonzalez-Usach et al. (2017) have presented SAFE-ECH - an IoT SOA system to monitor and control AAL residences. The system includes functionalities for the management of multiple residences, accessing globally and locally through an HMI. Diverse WSs enable communication to the HMI and a sensor observation service (SOS), complex event processor (CEP), and other services. The HMI allows authorized users to visualize information, configure system rules and services. The SOS manages sensor data, storing data observation based on the Open Geospatial Consortium semantics. The CEP includes rules to process SOS events and execute actions. Other services include an alarm manager to handle high-priority events and a broker to handle communication between system elements.

#### Centralized governance with distribution

Diverse projects maintain the governance in centralized components and use remote components (*e.g*., agents) to gather context, distribute services, or deploy processes. Smirnov, Levashova & Shilov (2011), with several authors (Smirnov & Levashova, 2009; Smirnov et al., 2010, 2011), proposed a WS context-aware decision support system for situation responses in emergency scenarios. It incorporates an ontological description of the resource network and situation management domain. The system includes profile management (*i.e*., capabilities of each operation member) for providing a role-based response. The system also comprises support services and agent-based services. The *support services* include core services (to manage context, registry, and monitoring) and operational services (to manage resources and decision-making), and wrap services for external sources. *Agent-based services* are intelligent agents collecting information, negotiating with other agents, and calling associated supporting services to execute action plans.

Amoretti (2012) have introduced an SOA framework based on autonomic hosts for AmI. The architecture consists of networked autonomic machines (NAMs) with ontologies to describe a smart environment. NAMs comprise resources and functional modules. The resources are fixed or changeable (*e.g*., battery level). The functional modules play the role (based on their definition) of context/service provider or consumer. Functional modules can also execute missions, functional policies, and self-management policies. The functional policies are mission-based processes (such as rules, algorithms, evolutionary plans). They enable a reactive service execution or call and publish context based on incoming events.

Pan et al. (2014) presented an SOA framework for AmI applications. It connects pervasive services through a bus capable of linking sensors and devices, software components, and distributed services (*e.g*., cloud, WS). The framework uses an ontology to represent heterogeneous services as uniform pervasive services. It includes a service bus that manages a group of sub-buses for effective peer-to-peer communication. A sub-bus operates services with similar protocols or frequently required. Sub-buses discover known protocols and register services (publish-subscribe pattern) on the main bus. The services are orchestrated with a planning-based approach based on (1) a task’s goal, (2) flow of services, and (3) context and quality requirements.

Nosović, Peters & Bruegge (2014) presented a framework for controlling smart environments, which abstracts device protocols providing consistent communication through an ESB which integrates: Device controller, device repository, client adapter, decision-maker, security controller, and environment tracker. The device controller provides the protocol abstraction. The device repository stores properties (*e.g*., protocol type, location). The client adapter provides a uniform request (*e.g*., get location) and set (*e.g*., set state). The decision-maker process rules (trigger events) and machine-learning (inhabitant recognition). The security controller provides authentication, access control, and priority-based requests. The environment tracker registers automatic state changes (*e.g*., temperature variation) or caused by users (*e.g*., turn on a light).

Prado, Ortiz & Boubeta-Puig (2017) presented CARED-SOA—an event-driven SOA for the IoT. It integrates a complex event processing (CEP) engine to provide a short-time data stream processing. CARED-SOA includes three components: ESB, alert manager, and context broker. The ESB facilitates communication. The Alert manager receives/retrieves data, adapts and stores it, and sends it to the CEP engine. Afterward, the CEP engine evaluates alert patterns and sends the IoT context (*i.e*., CEP matching data) to the context broker. The Context broker keeps a knowledge database of users’ context (*e.g*., age, location). Then, a context reasoner analyzes the context and sends notifications. Finally, a context adviser processes the notifications and sends them to the user. The authors improved the architecture to integrate client-side context (*via* a mobile app) and security functionalities (Caballero et al., 2021).

We presented in this section projects with remote components supporting service governance. They perform assigned tasks, but they maintain centralized governance in a central component, *e.g*., a complex event processing (CEP). Furthermore, remote components can also incorporate governance (*e.g*., collaborative policies), producing implementations that distribute governance. We present these implementations in the following section.

#### Distributed governance

Several approaches take advantage of remote components (*e.g*., proxies, gateways) close to sensors/actuators and locally manage resources and processes. In general, distributed governance optimizes the architecture by improving the performance for gathering context, integrating services, and providing remote user interaction.

Kawashima et al. (2009) have worked on a ubiquitous SOA platform for smart spaces, using distributed gateways to reduce overload over resource-constrained devices. The platform includes a server, smart space gateways, and smart applications. The server provides discovery, storage, service interface and management, communication, and sensing/actuation services. The smart space gateways connect heterogeneous devices for discovery and sensing. They support communication protocols and services to interact with devices or another gateway. Smart applications are both embedded applications in the server or web-based applications.

Bernardos, Tarrío & Casar (2009) have presented an SOA middleware to support AmI applications. It includes acquisition, fusion, presentation, and control layers. The acquisition layer gathers context from physical and virtual sensors. It implements software modules in gateways (on mobile devices) and signal adequacy and preprocessing in a server. The fusion layer implements services for feature extraction (through APIs) and an upper-level context inference. The presentation layer includes event notification services (context changes) to consumer applications. The control layer maintains persistence and enables a multi-layer registry, query, and subscription of services.

Reetz, Tonjes & Baker (2010) have presented an SOA solution to increase the performance of WSNs. It is based on the C-Cast middleware, where a central component – context broker – integrates different context providers connected to environmental, virtual, and logical sensors. The solution focuses on the environmental context provider, integrating wireless sensor gateways, and communicating with the context broker. The environmental context provider incorporates services. It also implements data management and sensor gateway interfaces for connecting the context broker, consumers, and other sensor gateways. Services include context detection and adaptation, sensor discovery and synchronization, data aggregation and fusion, and monitoring.

Roggen et al. (2011) presented Titan - an SOA framework for discovering resources based on user activity recognition. Titan consists of three components: a mobile device, Internet application repositories, and Titan nodes. The mobile device allows discovering resources from the personal area network, downloading code (if necessary), and executing services from pervasive applications. Internet application repositories store application references and application templates. It contains composite service graphs according to available resources. Titan nodes perform activity recognition; then, they instantiate, reconfigure, and execute sensor nodes’ services. They also provide communication, synchronization, management, and governance.

Degeler et al. (2013) presented an energy-aware SOA architecture for smart buildings. The architecture consists of three layers: physical, ubiquitous, and composition. The physical layer connects smart grid services (for pricing and local/external energy availability) and integrates devices using hardware gateways. The ubiquitous layer includes a context manager (for producing high-level context and activity recognition), repository manager (for maintaining data), and orchestration manager (for executing and scheduling actions). The composition layer includes control services (system interface, *e.g*., dashboards) and composition services (rule matching, AI planning, and computational fluid dynamics for heating conditions).

Stavropoulos et al. (2013) have introduced an SOA middleware for AmI. The architecture defines hardware, integration, and service and application layers. The hardware layer corresponds to physical devices such as ZigBee smart plugs, sensor boards, smart clampers, or Z-Wave devices. The integration layer consists of individual physical hardware drivers, including their interface, communication, and operation protocols. The service and application layers adopt a WSDL wrapping of device functions and complementary services, *e.g*., service to query IT context (*e.g*., ping, find MAC address, get CPU usage).

Buzeto et al. (2013) have presented uOS - a middleware for sharing resources in ubiquitous environments. Using proxies, uOS integrates different networks endpoints with diverse protocols (*e.g*., Bluetooth, WiFi) and resources (through a resource ontology). The architecture follows the Device Service Oriented Architecture (DSOA) and ubiquitous protocols (uP)—a lightweight set of protocols designed to facilitate communication in DSOA (Buzeto, Castanho & Jacobi, 2011). The middleware includes an abstraction of the software, hardware, and communication platforms using uP, removing constraints for integration. uOS combines network plugins, resource drivers, and applications. In addition, it includes a network layer (manages input/output and discovery), a connectivity layer (provides a message engine to manage uP protocols), and adaptability layers (integrates resources and applications).

Forkan, Khalil & Tari (2014) presented a solution for unifying context generation for AAL. It includes five cloud-oriented components: AAL systems, context aggregator and providers (CAP), service providers (SP), context-aware middleware (CaM), and context data visualization (VIS). The AAL systems comprise AAL cloud sensor data providers and service customers. The CAP abstracts context representation through context fusion and reasoning. The SP links applications or external services. The CaM processes, stores, and retrieves context and performs computational tasks such as management of services, context-to-service mapping, access control, and service delivery. The VIS provides a GUI for data (*e.g*., medical records). The solution includes an ontological context model, but the interoperability is through XML.

Fysarakis et al. (2018) presented XSACd—a smart environment framework for policy-based sharing and device services access. It adopts the eXtensible Access control Markup Language (XACML) to represent policies and the Device Profile for WS (DPWS) to specify resources as services. The architecture comprises five entities: Policy Enforcement Point (PEP), Policy Decision Point (PDP), Policy Administration Point (PAP) and Policy Information Point (PIP), Cross-domain Proxy (CP), and Broker. The PEP is on devices providing resources, allowing requests/authorizations. The PDP, which is on controlling nodes, evaluates requests/authorization. The PAP and PIP (on controlling nodes) create/manage policies and store attribute values. The CP enables routing discovery messages. The Broker distributes messages to subscribed proxies.

Dar et al. (2015) presented a resource-oriented architecture to integrate IoT devices into business process (BP) applications. It includes a registry of IoT services described with standard languages (*e.g*., WSDL) and an API to invoke IoT services. Then, a BP developer uses the API to compose IoT-aware processes. The architecture also includes an event manager for interaction (service subscription), a service replacement manager to query the registry, matching and selecting an appropriate candidate (or randomly selecting if multiple matches), and a device status monitor to register possible IoT device failures. A BP or the service replacement manager subscribes to update the failures. Multiple BP engines enable a distributed execution of processes in both a central server and smartphones.

Lee et al. (2017) have presented SoPIoT—an SOA IoT platform that abstracts as a service the functionalities of IoT devices and cloud. It allows registration of IoT device services using TCP/IP, lightweight protocols (*e.g*., Bluetooth) using gateways, and cloud functionalities as virtual devices. SoPIoT includes a script editor to compose services and middleware to store, monitor, and mediate service transactions. The middleware consists of three managers: (1) Device manager, for handling and monitoring devices; (2) Composite service manager, for translating scripts, monitoring, restarting (context-aware), and notifying service status; and (3) Data manager for sending data and checkpointing to perform recovery.

Malik & Kim (2019) presented a framework for sensor networks with geographical data management. It includes a virtual sensor platform to provide context (gateways connected to sensor networks) and a composite toolbox to create service profiles. The toolbox manages sensor networks with geospatial context, environment context, and physical sensor context. Service profiles are registered into a service registry, keeping available services at running time. The service profiles are then deployed to service platforms for service provision, querying the registry, and interacting through the gateways.

Pitatzis et al. (2020) presented a microservices-based platform for IoT devices available in AmI environments. The platform includes a gateway to provide service registration and discovery. The gateway connects IoT devices (physical or virtual things) microservices, *i.e*., independent building blocks/services handling its processes. It includes a rule engine to evaluate device rules with priorities. The platform handles policies to enforce proper device operation (*e.g*., can switch off only one minute after switching on) and provides a state maintenance mechanism to persist snapshots of devices during a shutdown to ensure service continuity.

Javed et al. (2020) presented a framework for smart cities to achieve cross-domain/cross-application service integration. The framework adopts open communication and data standards. It includes a service marketplace for data and service capability distribution and the corresponding functionalities and API to govern the services. The framework also proposes IoT gateways to integrate IoT systems/smart objects and external cloud-based solutions. The framework also adopts security functionalities (authentication, authorization).

Gooder et al. (2021) proposed a microservice-oriented middleware for service composition, matching device capabilities in smart environments. The middleware integrates IoT device functionalities to create on-demand services based on a service request. The middleware connects a service publisher and a service subscriber. The publisher gathers IoT device data/capabilities and make them available to the middleware. The subscriber interacts with the environment, *e.g*., to manage user service requests. The middleware matches the capabilities and requests, assembling and delivering a composite service. The middleware also includes security/auditing functions and QoS monitoring.

Architectures with distributed governance involve similar technologies/languages/protocols as centralized governance (*e.g*., WS, ontologies). The difference lies in the resources, capacities, and policies of the distributed components (*e.g*., mobile devices, brokers). However, still exist a centralized component (*e.g*., WS server) that provides the governance. Conversely, when this centralized component is indistinguishable among distributed components, the governance depends on the behavior of all components (*i.e*., intelligent agents).

#### Governance including intelligent agents

Diverse approaches implement agents with rules to represent complex behavior. Even though the following projects also include non-agent components, this section focuses on the governance implementation in agents, *e.g*., agents performing discovery/composition of services.

Soldatos et al. (2006) built a framework for integrating perceptual components and the context of space, creating a model of the situation, thus enabling intelligent resource discovery and management. It includes an ontology knowledge base (KB) and three layers: sensory, perceptual, and agent layers. Sensor proxies manage the dynamism of sensing the environment. The perceptual layer is implemented using IBM’s CHILIX library, enabling person localization/tracking, body detection/recognition/tracking, speech/acoustic/emotion/activity recognition, and lips observation. The agent layer includes agents for discovering and registering services in the KB. It also manages device integration, user profile, and service requests and performs system management and monitoring.

Miyata, Morikawa & Ishida (2009) have worked in a service-oriented smart space learning system. The architecture (Suo et al., 2008; Miyata, Morikawa & Ishida, 2009) is based on WS and includes three components: WS wrapper agent (WSWA), smart platform agent web service (SPAW), and open smart platform gateway (OSPG). WSWA is an intermediary to external services (*e.g*., language services), invoking them as required by other agents. SPAW is the WS server, providing interaction inside and outside the platform and creating agents to manage requests. The OSPG is a proxy for mobile devices, allowing interaction with platform services using a web browser.

Bhuvaneshwari & Sujatha (2012)⁠ have worked on VAISTC4 - an agent-based SOA system for traffic congestion control. It consists of a backbone of agents merging information from different sensors (*e.g*., cameras), preprocessing the data, and putting relevant information in a database. VAISTC4 is based on the ATRACO framework (Goumopoulos et al., 2008; Seremeti, Goumopoulos & Kameas, 2009; Bidot, Goumopoulos & Calemis, 2011), *i.e*., an SOA middleware for AmI systems. ATRACO includes an ontology model for the user and environment profiles (Seremeti, Goumopoulos & Kameas, 2009) and allows service composition through AI planning and workflow (Bidot, Goumopoulos & Calemis, 2011). In addition, VAISTC4 provides event channels for registering sensor and effector agents. Sensor agents are attached to devices and can command events (*e.g*., change the timing of a traffic light), and effector agents are scattered in the environment close to users (*e.g*., mobile).

Familiar, Martínez & López (2012) have presented an architecture for wireless *ad hoc* and sensor networks that includes three platforms: physical device, pervasive, and SOA. The physical device platform abstracts the hardware and provides software capabilities for integrating devices. The pervasive service platform uses the separation of concern paradigm to identify and encapsulate properties into different services. The SOA platform constitutes the backbone of the integration. It includes a control service layer (based on a publish-subscribe approach (Familiar et al., 2012)), cross-layer services (providing security and resource-available-service reasoning), low-level service layers (agent-based components and context discovery), high-level service layers (service control and QoS), and internetworking service layers (agent-based service composition).

Tapia et al. (2013), together with several authors (Corchado, Tapia & Bajo, 2012; Tapia, Fraile & Rodríguez, 2009; Tapia & Alonso, 2010; Tapia, Alonso & Corchado, 2011)⁠, have worked on a solution for an agent and SOA integration of heterogeneous WSN. It includes a multi-agent layer (called FUSION@) and remote service-based agents (called HERA). FUSION@ implements agents and communication capabilities to facilitate the distribution and management of resources and services, allowing for moving functions to where actions are required. HERA is directly embedded in WSN to manage sensing data and is the evolution of SYLPH – the first SOA platform with an application layer and a message layer (Tapia & Alonso, 2010; Tapia, Alonso & Corchado, 2011). The platform includes an admin, an interface, a supervisor, and security agents with explicit policies to support the execution.

Ferilli et al. (2015) presented a smart environment MAS architecture to execute services for actions in user workflows. It comprises a knowledge base (KB) and three layers: environment, reasoning, and learning. The environment represents sensors, actuators, and services. Reasoning and learning layers are agent-based with the following modules: (a) AmI coordinator (applies the KB), (b) incremental theory learner from examples (refines the KB), (c) workflow manager (controls executions), and (d) service planning identifier and composer (provides service composition based on semantic Web). A composite service interoperates with the workflow manager to achieve goals based on constraints, preferences, or requirements. Then, the workflow manager can act (through agents) or refine a process (learning).

Fysarakis et al. (2015) have presented a framework for real-time security, privacy, and dependability of AmI systems based on pre-defined metrics. It includes four layers: node (embedded devices), network (connected nodes), middleware (network management), and overlay (control agents). The framework uses the OSGi platform for SOA compliance and management of embedded devices. Different agents are deployed into OSGi, implementing two core components: reasoning and ambient/system manager. The reasoning evaluates the metrics and composes elements (*i.e*., operations, attributes) from the different layers. Afterward, in real-time, the ambient/system manager controls the resulting composition, *e.g*., change the configuration to increase the security level.

Mohamed et al. (2017) have presented SmartCityWare—a middleware to integrate cloud and fog computing for smart city services. It includes a service layer and a multi-agent runtime environment. The service layer provides core services and environmental services (*e.g*., cloud, fog, IoT services). The core services enable a secure and location-based invocation of services. It includes a broker that is responsible for environmental services advertisement, discovery, and registration. The multi-agent runtime environment includes agents to deploy, schedule, and support the execution of distributed services and provide governance of available fog resources.

Architectures that include agents provide a level of “intelligence” because they act based on their policies/rules. However, the agents cannot control unknown situations (*i.e*., situations not contemplated in their rules). Some projects propose autonomic computing/autonomous agents with a self-governance of services for governance in unknown situations.

#### Self-governance: governance in autonomic/autonomous architectures

Self-governance is a challenging aspect of SOA projects. It involves a higher level of adaptation in service processes (*e.g*., discovery, composition) for adjusting them to the situations. They include a knowledge base that provides feedback on the situations and learning processes and improves adaptation through changeable policies/rules.

Kim et al. (2006) introduced an SOA ubiquitous function (UF) model for the smart control of robots in AmI. UFs represent the environment and its capabilities, allowing operations through functions. The architecture includes three main services: smart object, discovery, and logic. The smart object manages physical objects, mapping them to UFs. The smart discovery finds and integrates UFs. The smart logic controls robots’ mission, combining objects with sensors and actuators. It is based on a self-adaptive discovery, registry, and combination of UFs, using a robust internal-loop compensator (RIC) – a multiple feedback logic controller with an internal-loop to find and compensate for disturbances (*i.e*., obstacles for the robot’s mission).

Penserini et al. (2010) have described a model for service-oriented organizations (SOO) of autonomous agents. The SOO combines service-oriented computing matchmaker and broker agent patterns (Fuxman & Giorgini, 2001; Bresciani et al., 2004a) with Tropos—an agent-oriented software engineering methodology for organizational modeling (Bresciani et al., 2004b; Penserini & Bresciani, 2005). The model includes three SOO architectures: matchmaker, broker, and implicit. The matchmaker and broker SOO extend matchmaker and broker agent patterns, respectively. Both support the provider organizer role with either a consumer initiator (matchmaker) for a direct execution provider-consumer or through an intermediary (broker). The implicit SOO discards the pre-defined provider organizer role. At runtime, a winner provider assumes the organizer’s role. It coordinates services, enabling faster environment support.

Chun et al. (2011) have presented a self-adaptive scheme for smart space services, using context history as an additional soft-feedback adaptation. It includes an adaptation agent and adaptable software. The adaptation agent gathers context data from sensors. The adaptable software handles internal adaptation using collected data such as sound, seismic, light, video, audio, temperature, and system data such as processes, CPUs, and memory use. The adaptation process follows adaptation plans, including policies and rules. When the system detects the need for a strategy, it executes an adaptation plan—using learning algorithms, the system stores both performance and the executed plan in a knowledge base.

Acampora, Loia & Vitiello (2011) have presented an SOA autonomous multi-agent framework for being aware of a user’s emotional condition for providing comfort services in AmI. It includes three layers: sensor network, cognitive multi-agent system (MAS), and SOA platform. The sensor network captures users and surrounding contexts. The cognitive MAS manages the distribution of emotional services in the environment. The SOA platform provides service interaction, improving the user’s comfort, *e.g*., soft or hard music services, volume control service. The system adapts its conditions based on the human mood’s representation, including variation in emotional, environmental, and temporal situations.

Abdulrazak et al. (2011) presented ContextAA—a self-organized service architecture for micro context-awareness of distributed agents, perceiving their local environment, and acting based on their roles. Micro context-awareness represents the usual categories of context (*e.g*., activity, identity, location, time) and operations and entities such as publishing, requesting, and obtaining an ontologically meaningful subset of contexts for performing a service in a given agent. The architecture (Abdulrazak et al., 2011; Roy, Abdulrazak & Belala, 2011) includes host components, agents, context, and context space. Host components serve as middleware, interacting with others and integrating external services. Agents are context-dependent entities responsible for executing actions, differentiating between standard (host services) and user (or domain-specific) agents. Context and context space are organized knowledge repositories at both the agent and host levels.

Ruta et al. (2018) presented a semantic-based framework for home and building. It defines autonomous device agents that interoperate to share and orchestrate resources. First, an agent describes its basic features (device type, location, hardware) and its services, such as the device configuration. Next, agents can interact and “post” context, exploited by other agents for sensing (context/events) or actuating (*e.g*., request for changing a configuration). Agents then actuate or start a discovery process to find potential agents providing suitable services. The semantics follows the Linked Data Platform W3C recommendation, defining devices, services, posts, and other semantic descriptions.

The previous architectures involve the use of context as a key aspect of improving governance. For example, context history and cognitive agents can enhance the governance of services and, as a result, service delivery. On the other hand, an advantage of autonomic/autonomous architectures is that computers can reach desired entity capabilities by self-governed policies/rules, providing self-service.

### Mapping and classification

**Figure 3:**
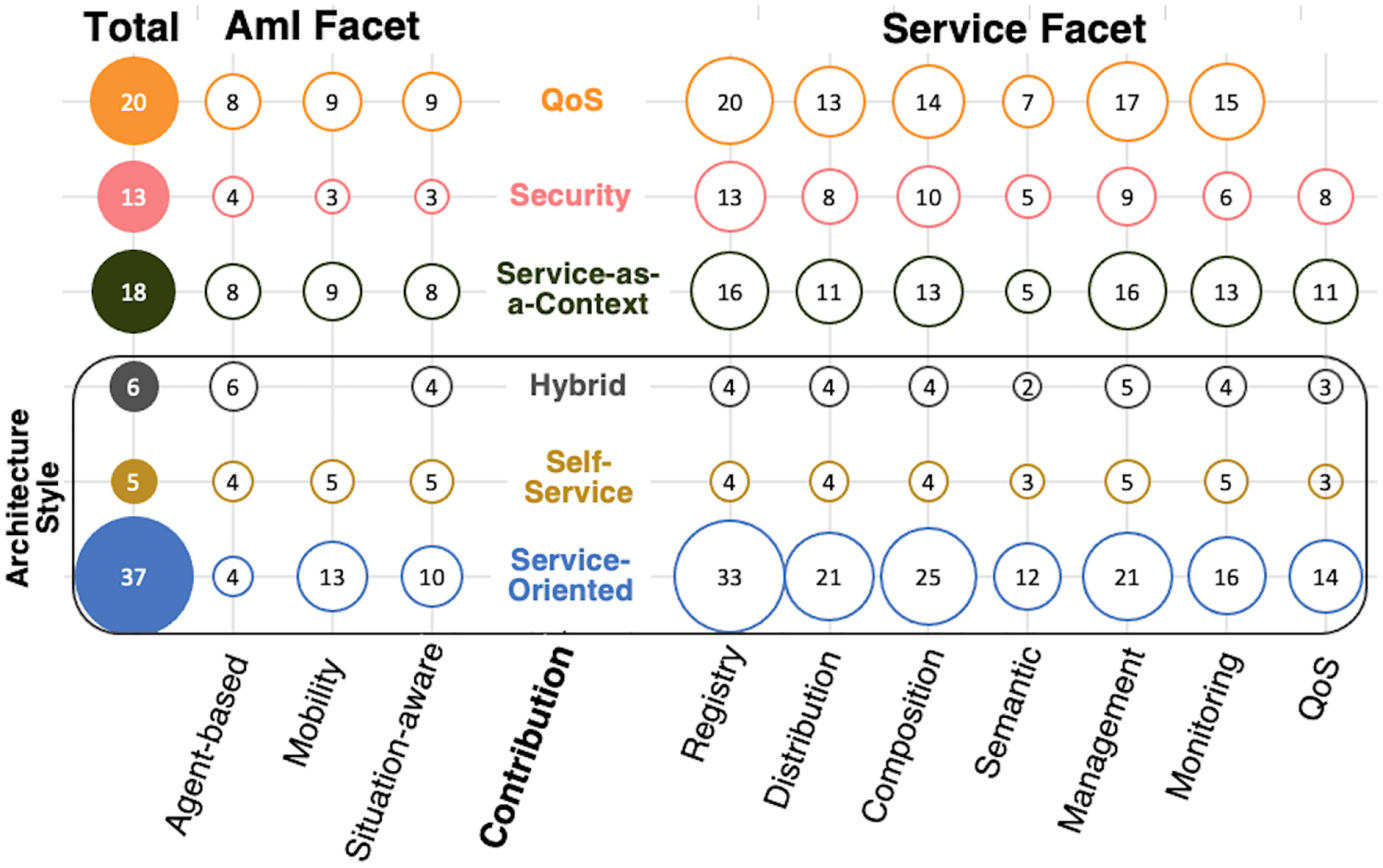
Systematic map (contribution per characteristic).

Following the analysis, we classified the papers (Tables 6 and 7). Figure 3 summarizes the research efforts considering each quantifiable characteristic of the classification scheme. In the figure, the contribution axis represents orthogonal aspects analyzed in this survey, inter-relating service-oriented, AmI, and research/global facets. We also considered the quality of service (QoS) in the contribution axis to analyze QoS to AmI elements, *e.g*., the context history in Chun et al. (2011) and the quality of space in Gouin-Vallerand et al. (2010).

The registry is the service feature most considered among the research efforts with 39 projects (31 are service-oriented, four are self-service, and four are hybrids). Semantic interoperability, which refers to explicit semantics, is the least applied to 16 projects. However, some projects’ interoperability use implicit semantics inherited from SOA, *e.g*., Pitatzis et al. (2020) adopts REST semantics. We classify projects with implicit SOA semantics as syntactic interoperability (*i.e*., no semantics at the project level). The research efforts adopt other characteristics on different levels. In the AmI facet, 13 projects are agent-based, 18 incorporate architecture components on mobile devices, and 19 propose a higher-level adaptation (situation-aware), introducing state, history, relevant context (*e.g*., Acampora, Loia & Vitiello (2011) consider human emotions for context awareness).

The reviewed solution proposals (Table 6) are domain-specific projects, and thus, they mainly consider the required features for implementation. For example, Paganelli & Giuli (2011) propose a healthcare support system at home that integrates the situation of the patient (*e.g*., actual activity) and heterogeneous sources (*e.g*., bio-signals and environmental context) for a situation-aware adaptation, regarding semantic interoperability, in a centralized WS with static access to the services.

## Results and discussion

According to the systematic map process, we detail in this section the analysis, summary of information per facet, and comparison of characteristics using tables and graphs to answer the research questions. Following, we highlight the relevant aspects of the selected projects.

### Ambient intelligence facet

Research efforts provide solutions for various application domains, including, among others, smart home/building, assisted living, healthcare, wireless sensor networks, IoT, and applications targeting business and the city (Table 6). Furthermore, several projects target a framework/model general applicable to smart space or AmI fields. We found a few projects applying security to distributed agents or mobile components inside the domain or field. For example, 18 projects propose mobility (*e.g*., components in mobile devices), but only three propose a security mechanism in their design. Similarly, 14 projects consider agents (seven agent-based and seven with agents as part of the architecture), but only four propose security.

**Figure 4:**
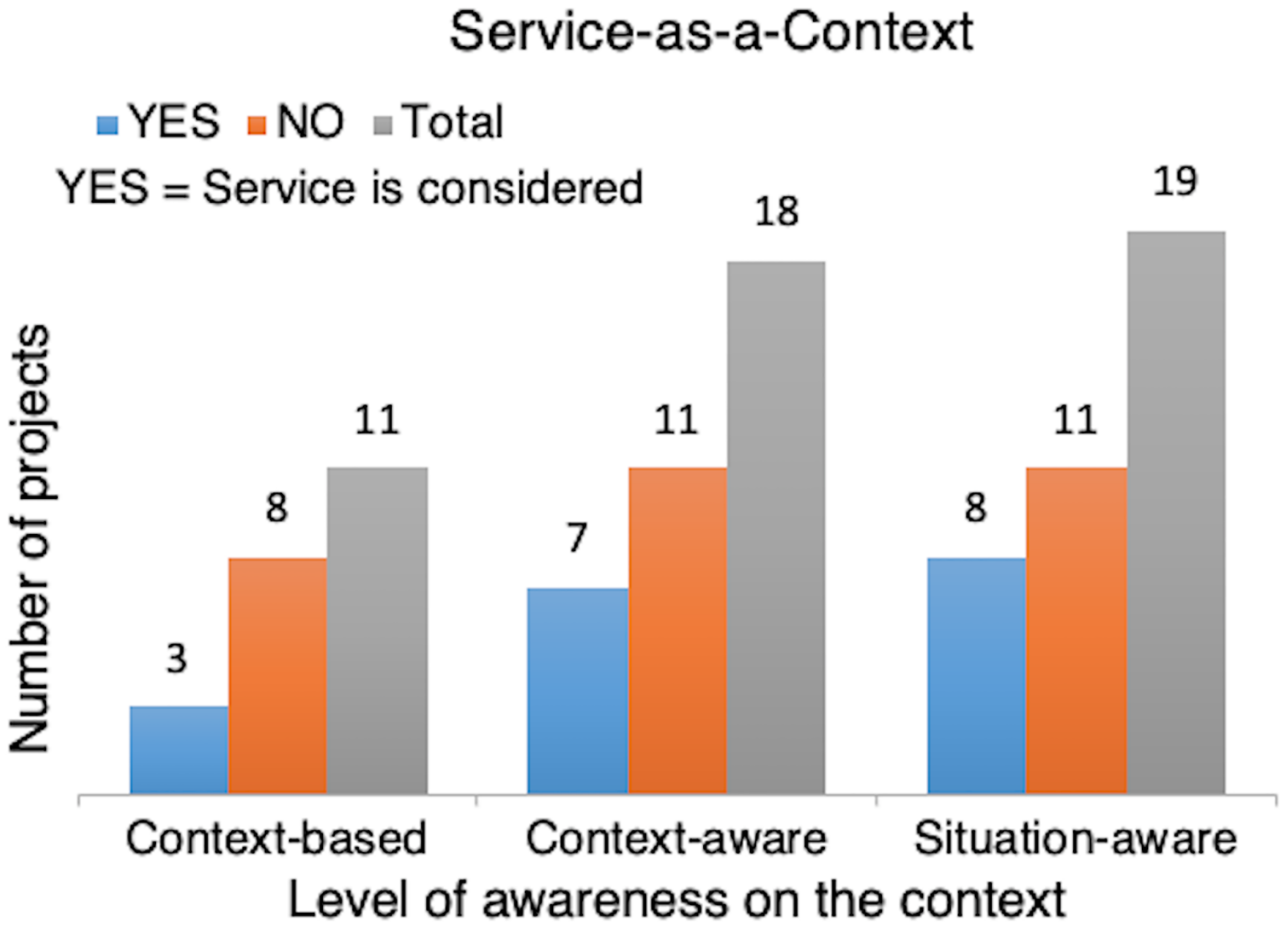
Awareness of the context and service.

Also, several projects incorporate the service provided in the context-awareness process to improve adaptation (Fig. 4). Eight projects out of 19 consider service-as-a-context when the application demands a higher adaptation (situation-aware). This ratio is lower in context-aware adaptation, seven out of 18, and for context-based adaptation, it is three out of 11 (Fig. 4).

### Service facet

**Figure 5:**
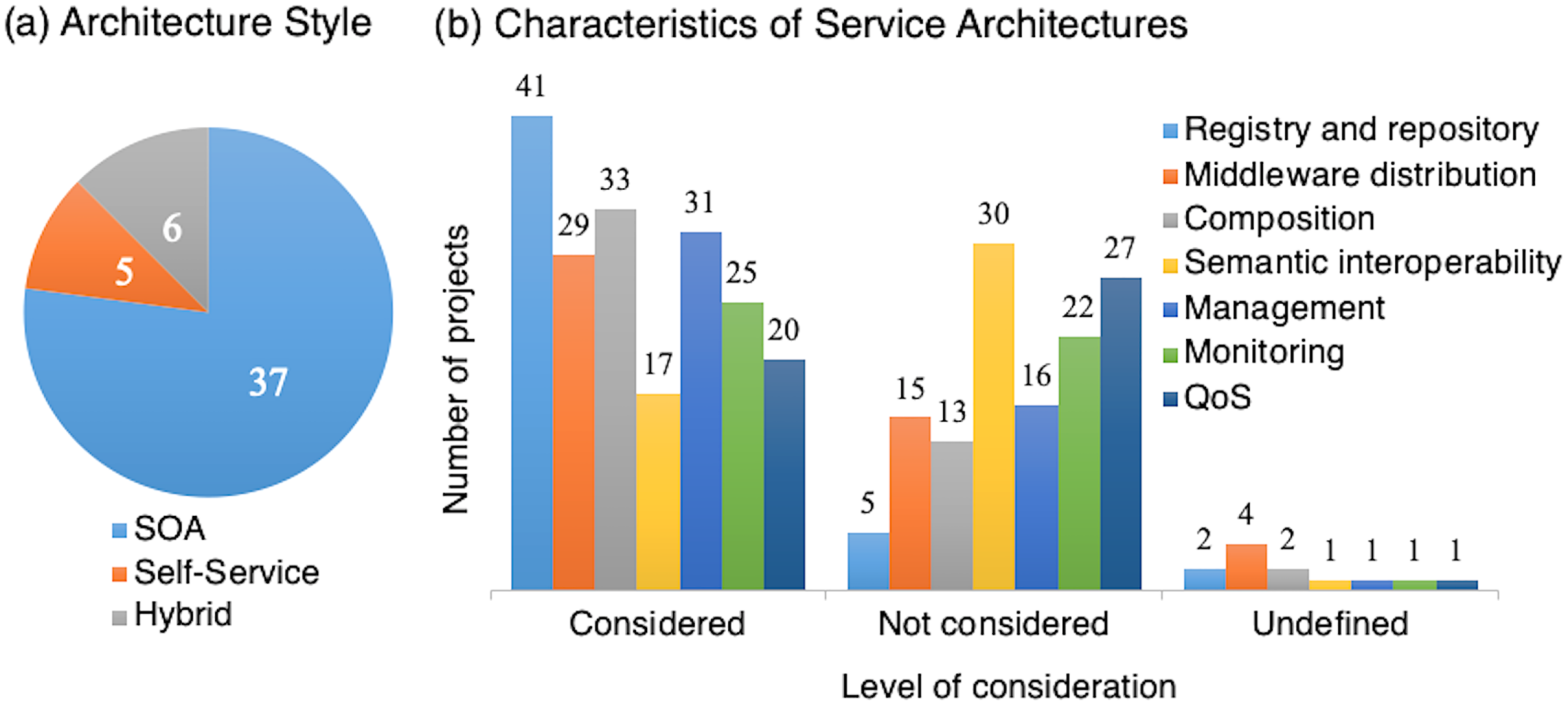
(A) Projects by service architecture. (B) Distribution of SOA aspects.

Figure 5 shows the distribution of service-oriented aspects. Figure 5B compares the distribution where the aspects are either not considered or undefined. The prevalent architecture is SOA-based, with 37 projects following the SOA model, implementing diverse governance (Table 7). Five projects extend the SOA model to the autonomic computing paradigm proposing self-service architectures, and six projects present hybrid architecture. The projects apply different techniques for each aspect of SOA, *e.g*., lookup, subscribe, direct access, and querying the registry for discoverability (Table 7).

### Research and global facets

**Figure 6:**
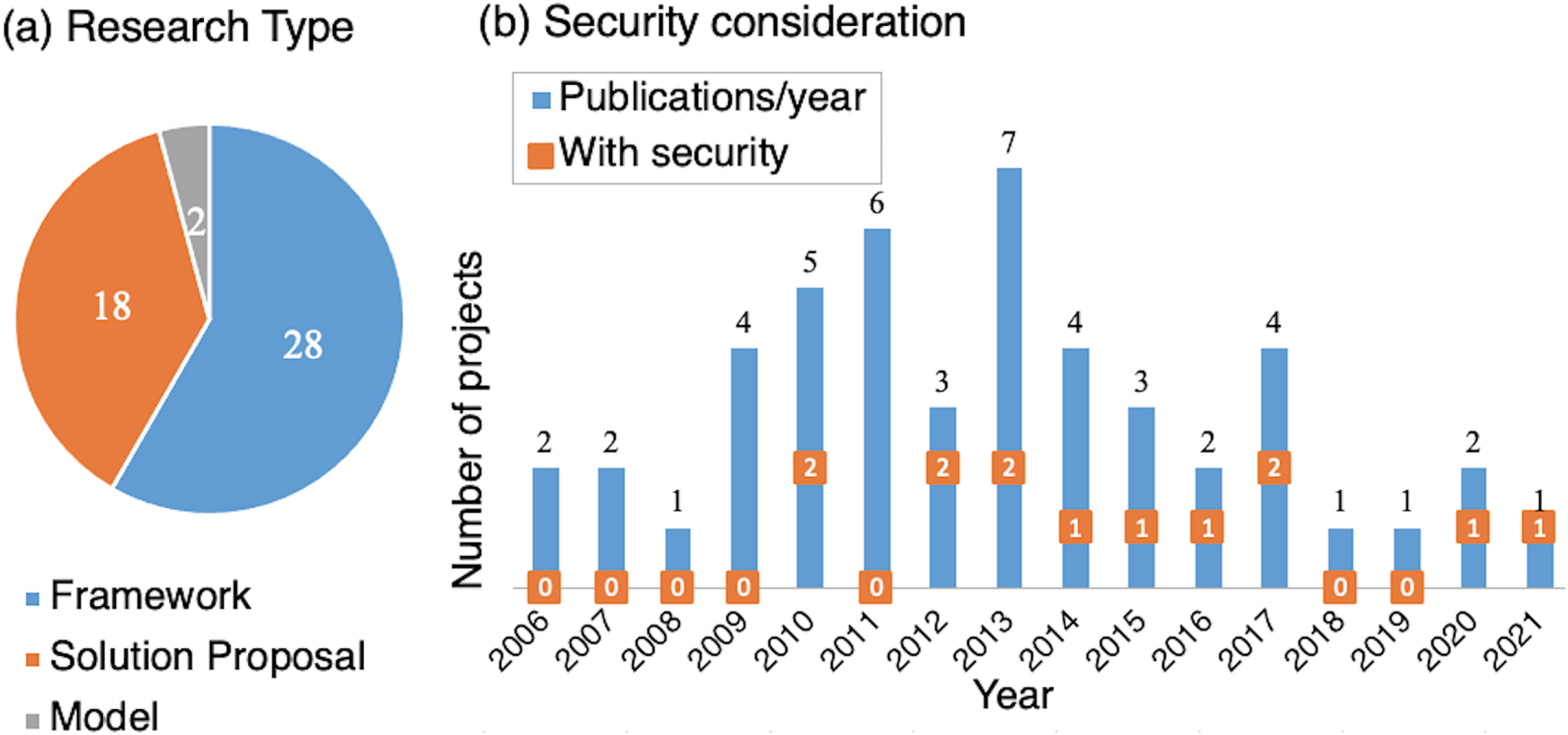
Projects by (A) research type, (B) year and consider security.

The demand for ambient service support systems is present in both domain-specific applications and general designs. Therefore, we analyzed the target of the research, finding 18 projects proposing a solution in an application domain (*e.g*., assisted living); 28 projects proposing a framework (domain-specific or general); and two projects proposing a general-purpose model (Fig. 6A). On the other hand, few projects considered security mechanisms (*e.g*., access control): Two in 2010, 2012, 2013, and 2017, and one in 2014, 2015, 2016, 2020, and 2021 (Fig. 6B).

### Limitations of the study

The central focus of the survey is to analyze the SOA model in AmI implementations. Therefore, we refined the research queries with explicit exclusion of either the AmI or the SOA concepts. The survey also disregards specific aspects such as representing the context, AI algorithms for reasoning, or interaction styles between entities (Table 1 lists other aspects). We also discarded general adoption research efforts with no details of SOA/AmI applied characteristics. As an example of the general case of the discarded papers, we can find WS applications such as regulating the temperature for healthcare support without a registry of services, monitoring, QoS. The limitation is that these discarded projects could contribute to new approaches such as augmented adaptation (*e.g*., social-aware).

### Open challenges and research directions

Distributed computing, mobile systems, and the Internet of Things (IoT) marked the last two decade’s development of ***ambient intelligence*** (Zhao & Wang, 2010; Lin et al., 2017). New technologies, such as cloud/edge computing and 5G, provide connectivity to massive IoT surrounding devices in multiple settings (Pham et al., 2020). As a result, ubiquitous technologies are available to interact with ***users*** who exploit resources through **context-aware** applications.

Our analysis enabled us to highlight the research challenges and trends of ambient intelligence systems. A couple of traditional challenges have been presented in diverse reviews, *e.g*., constraints in resources for running services, scalable IoT discovery, protocol interoperability, and security (Perera & Zaslavsky, 2014; Issarny et al., 2016). Following, we present our identified challenges and research directions.

#### Intelligent ubiquitous services

Computer support is becoming indistinguishable (Weiser, 1991). Researchers are proposing systems for decreasing the cognitive load on users (Weiser & Brown, 1996), systems for pushing technology in moments of cognitive rest (Hallnäs & Redström, 2001), and context-aware systems (Schilit & Theimer, 1994; Abowd, Dey & Brown, 1999). In addition, advances in artificial intelligence and hardware technology contribute to services that appear when necessary.

However, it is still necessary to provide services that vanish in everyday activities and objects. Therefore, different technologies and mechanisms direct the research for providing intelligent ubiquitous services, *e.g*., body sensors gathering vital signs inputs to the AI algorithm in health care systems (Acampora et al., 2013), sensing-as-a-service QoS model for managing billions of sensors in the IoT (Perera & Zaslavsky, 2014), distributed sensing and big data for an accurate mobile context-awareness in cloud computing (Rahimi et al., 2013). In particular, these research directions have to involve learning and further mechanisms to understand the person (psychological-aware) (Bao et al., 2013), provide individual (in-person) augmentation (Xia & Maes, 2013), anticipate the intentions (anticipatory adaptation) (Pejovic & Musolesi, 2015; Pejovic & Musolesi, 2014) and strengthen the service architecture, *e.g*., implementing recovery services.

#### Self-governance: autonomic service providers

The concept of autonomic computing (Kephart & Chess, 2003) continues materializing, bringing self-service and hybrid architectures to AmI. An emerging framework is the open smart environment (Abdulrazak et al., 2011) which extends the support system to the person’s space, providing personal assistance in mobile, distributed, and dynamic scenarios. Open smart environments release the controlled architecture by implementing agent-based support and enabling the use of the context as a pervasive self-governing service provider (Gouin-Vallerand et al., 2008; Abdulrazak et al., 2010b, 2011). Agents as service providers describe the user and the system’s situation in their context, containing self-descriptions of their capabilities and existence.

Service interoperability and distribution are complex aspects of governing autonomic architectures for enabling inter-system interoperability^4^ 4Between systems, interoperability is the ability of two or more systems or components to exchange information and use the information that has been exchanged (IEEE Standard Computer Dictionary).. Self-governed service providers maintain knowledge of the self, being aware of their capabilities but restricted, conscious of the global situation among entities. Specifically, the autonomic architecture has to manage conflicts between entities, integrate legacy systems, the federation of services, and implement community-based service conflict resolution. Maintaining a global quality of service (QoS) in autonomic service architectures is challenging. It requires QoS definitions into learning and artificial intelligence techniques to achieve self-adaptation (Razzaque et al., 2016).

#### Empowering people

Non-[ICT]-technical people use applications in AmI, *e.g*., solution proposals in the survey (Table 6). Even though SOA contracts can be human readable, *e.g*., using plain text or XML, non-technical people encounter difficulties managing services. Still, AmI systems use different technologies and data formats, bringing on the necessity of technological support. Nowadays, domain experts use tools for their activities (*e.g*., using a business process management engine (Baresi, Guinea & Pasquale, 2007; Albreshne, Lahcen & Pasquier, 2013)). These tools require technical support, preventing users from creating their services, allowing only pre-existing components and services.

Other approaches facilitate the integration of context from service providers to support users. For example, IFTTT (https://ifttt.com, last accessed 2021-06-30) allows for the creation of condition→action rules (called recipes) from pre-defined services available as building blocks (called channels). Other platforms simplify building complete applications. *E.g*., Apache Cordova (http://cordova.apache.org, last accessed 2021-06-30) allows building mobile applications for different mobile platforms based on web standards, integrating the device’s sensors and external services through plugins. However, when providing this service, these approaches only consider device context, disregarding user profile. A service can produce a different significance for various users or multiple significances for a user, *e.g*., a service for a comfortable temperature in a room for a healthy user and a user with the flu.

An emerging approach is end-user as application builders, using cognitive-aid metaphors and software engineering techniques (Paternò, 2013; Burnett & Myers, 2014). End-user software engineering introduces techniques into users’ existing workflow, managing the unplanned, implicit, opportunistic, instinctive, and self-priority intents over their applications regarding quality concerns (Ko et al., 2011). These techniques combine artificial intelligence, cooperative work, and human-computer interaction (HCI) patterns to facilitate application development by end-users (*i.e*., non-technical people) (Lieberman et al., 2006).

#### Security

Security and safety are constant research topics. Most surveys describe security and privacy issues as challenges. Safety and ethical issues are also identified, especially for healthcare systems. Our study found 13 out of 48 projects considering security aspects regarding (a) context security (*e.g*., encryption) and (b) security functionalities such as authentication and authorization for using services. In general, a primary aspect is to provide an appropriate integration of heterogeneous resources and maintain adequate security in communication mechanisms and protocols such as Wi-Fi and Bluetooth. Similarly, available services on the Internet increase the context for AmI/IoT applications, *e.g*., Amazon Alexa skills (*i.e*., services to integrate) have grown to more than 40.000 in 2018 (Kim & Kim, 2018). These services, often implemented by third-party developers, are a potential security risk.

In a controlled SOA infrastructure (*e.g*., centralized servers, cloud), it is feasible to deploy security algorithms and mechanisms. Autonomic self-service also defines policies for self-protection, but security is challenging. The emerging paradigm Fog computing enables the distribution of services but introduces diverse challenges such as access control and encryption key management (Alrawais et al., 2017). Blockchain technology, capable of achieving decentralized/autonomous security, is an open research topic in fog/edge computing (Omoniwa et al., 2019). Also, a security challenge is in society, particularly in integrating legacy and external systems and implementing federated services.

## Conclusions

Enabling technologies, resources, and services for Ambient Intelligence applications is diverse—all physical or virtual things could be regarded as context. With ubiquitous technologies and IoT, ambient capabilities increase, making available even more resources and services. Due to the diversity of resources in environments, effective governance of these resources is required for supporting users. A common approach to govern resources in ambient intelligence systems is service-oriented computing. In this approach, services represent the capabilities of entities, and a service platform provides the governance of the services. We presented in this paper the ***Ambient Intelligence Perspective*** and the ***Results of our review and analysis*** of Ambient Intelligence system implementations considering service-oriented architecture (SOA) as a reference model for the distribution and delivery of AmI services.

### Ambient intelligence perspective

Ambient Intelligence is one of the paradigms of computer science targeting automating the interaction with surrounding resources. New computation technologies, such as cloud computing and edge/fog computing, make available computational services and control everyday objects and devices. These technologies augment ambient intelligence systems through context-awareness. They sense and actuate *via* physical artifacts such as IoT devices (*e.g*., lights) in a dynamic and distributed setup (*e.g*., street lights). Applications in diverse domains adopt ambient intelligence to provide ubiquitous services involving context-aware development.

Ambient Intelligence extends the boundaries of controlled architectures for supporting users, maintaining an efficient response to end-users based on available resources and services everywhere. The new generation of AmI applications is augmented with artificial intelligence, combining resources in the ambient, covering possible situations, and strengthening human interactions. A complete ambient intelligence achievement is still a research challenge; however, AmI is further considered for different domains and indoor/outdoor settings. Thus, the AmI governance is becoming an essential aspect of managing changes and system components such as available resources/services, user dynamicity, and diverse situations.

Large-scale Ambient Intelligence deployment is a continuous industrial and research effort. It can be achieved when computing systems can provide all services for continuous support, matching available resources by extracting functional characteristics from deployed applications, and running situation-aware processes in ambient components (*e.g*., self-governed agents) that provide and adapt environments for user support.

### Review overview

This paper presents a systemic situation in AmI systems to identify common service-based features, including what type of contexts are used and whether the applications consider the service itself as a context. We also reviewed different architectures and how they manage the quality of service, governance, and security. We applied a systematic mapping process in our study by defining a categorization schema for analyzing existing research projects. The categorization supplies characteristics for an objective classification based on concepts and existing surveys. We also defined three types of service architecture styles for AmI (following the SOA model): (i) SOA-based with a central server or cloud, (ii) self-service with autonomic computing, and (iii) hybrid. Furthermore, we defined five groups related to the governance design within the architecture style (*i.e*., SOA-based, self-service and hybrid): (a) centralized governance, (b) centralized governance with distribution, (c) distributed governance, (d) governance including agents, and (e) governance in autonomous architectures with self-governing policies.

In this study, we have highlighted the diversity of the projects adopting SOA in AmI, from a smart house to a smart city, including the several projects that extended QoS by including the situation and user-profile to improve the service provisioning. We have also identified the challenges of AmI service systems: (a) For the service provider, it is necessary to enhance the learning and proactive support as well as delivery mechanisms to guarantee the continuity of the service provided by autonomic services; (b) For the client, it is essential to empower non-technical people to use the available pervasive services.

Furthermore, emerging technologies, such as the IoT, are enabling pervasive services. Therefore, it is necessary to correlate the categorization scheme and the characteristics of emerging technologies (*e.g*., self-organization of IoT devices) to produce an aggregation of attributes to evaluate the AmI systems’ evolution.

## Additional Information and Declarations

### Data Availability

The following information was supplied regarding data availability: This is a literature review, the data appears in Tables 6 and 7.
